# Lysogenization of *Staphylococcus aureus* RN450 by phages ϕ11 and ϕ80α leads to the activation of the SigB regulon

**DOI:** 10.1038/s41598-018-31107-z

**Published:** 2018-08-23

**Authors:** Lucía Fernández, Silvia González, Nuria Quiles-Puchalt, Diana Gutiérrez, José R. Penadés, Pilar García, Ana Rodríguez

**Affiliations:** 1 Instituto de Productos Lácteos de Asturias (IPLA-CSIC), Paseo Río Linares s/n 33300 -, Villaviciosa, Asturias Spain; 2 Institute of Infection, Immunity and Inflammation, College of Medical, Veterinary and Life Sciences, University of Glasgow, G12 8TA Glasgow, UK

## Abstract

*Staphylococcus aureus* is a major opportunistic pathogen that commonly forms biofilms on various biotic and abiotic surfaces. Also, most isolates are known to carry prophages in their genomes. With this in mind, it seems that acquiring a better knowledge of the impact of prophages on the physiology of *S*. *aureus* biofilm cells would be useful for developing strategies to eliminate this pathogen. Here, we performed RNA-seq analysis of biofilm cells formed by *S*. *aureus* RN450 and two derived strains carrying prophages ϕ11 and ϕ80α. The lysogenic strains displayed increased biofilm formation and production of the carotenoid pigment staphyloxanthin. These phenotypes could be partly explained by the differences in gene expression displayed by prophage-harboring strains, namely an activation of the alternative sigma factor (SigB) regulon and downregulation of genes controlled by the Agr quorum-sensing system, especially the decreased transcription of genes encoding dispersion factors like proteases. Nonetheless, spontaneous lysis of part of the population could also contribute to the increased attached biomass. Interestingly, it appears that the phage CI protein plays a role in orchestrating these phage-host interactions, although more research is needed to confirm this possibility. Likewise, future studies should examine the impact of these two prophages during the infection.

## Introduction

Over the last 100 years, we have been gradually discovering the impact of bacteriophages, viruses that infect bacteria, on the fate of microbial communities^1,2^. In the case of prophages and prophage-like elements, for instance, advances in genome sequencing have revealed that phage-like sequences are present in almost all bacterial genomes and that they can amount to as much as 20% of the genetic material in some bacteria^3^. This fact indicates that phages have successfully spread within bacterial populations, and hints that carrying phage-related DNA in the bacterial chromosome might confer evolutionary advantages to the host. One of the most widely accepted beneficial roles of functional prophages is that they make the host bacterial population more competitive against phage-sensitive strains^4^. Thus, spontaneous prophage activation and subsequent lysis of individual cells within the population will release viral particles that can infect and lyse cells of the susceptible strain. Furthermore, the intracellular cell contents of the lysed cells can then be used by other cells as nutrients^5^. Conversely, other studies have pointed out that lysogeny may be detrimental under certain conditions. A clear example is the inhibition exerted by *Streptococcus pneumoniae* against *Staphylococcus aureus* in the nasal cavity. An elegant study by Selva *et al*.^6^ demonstrated that this lesser competitive ability was due to the induction of the lytic cycle in *S*. *aureus* lysogenic strains by subinhibitory concentrations of H_2_O_2_ released by *Streptococcus*, a trigger of the SOS response. Nonetheless, the effect of prophage carriage on bacteria goes well beyond competition and can have a profound impact on the exchange of genetic information as well as diverse physiological traits. Regarding horizontal gene transfer, phages are known to participate in the dissemination of virulence and/or antibiotic resistance markers^7^. For instance, the Shiga toxin in *Escherichia coli* and many *S*. *aureus* toxins are harbored by prophages^8^. Additionally, bacteriophages help mobilize the superantigen-encoding pathogenicity islands (SaPIs) of *S*. *aureus*^9^. Prophages can also affect the fitness of their bacterial host. Thus, some cryptic prophages can increase the growth rate in *E*. *coli*^10^, whereas *Bacillus thuringiensis* strains lysogenized by tectiviruses GIL01 and GIL16 showed a lower growth rate than their non-lysogenic counterparts. *In B*. *thuringiensis*, lysogenic strains also displayed greater swarming motility and reduced sporulation rate^11^. Another widely studied phenotype in lysogenized strains is biofilm formation. The results obtained so far indicate that there is no general rule in this regard, with some phages promoting dispersal, as is the case of prophage-harboring *Enterococcus faecalis* V583ΔABC^12^, while others enhance the development of sessile communities. An example of the latter is the environmental bacterium *Shewanella oneidensis*, in which bacteriophage-mediated lysis contributes to the release of eDNA to the extracellular matrix and, consequently, promotes biofilm development^13^. In *Pseudomonas aeruginosa*, the filamentous prophage Pf4 plays a fairly complex role in biofilm development and is essential for microcolony architecture^14,15^.

The human and animal pathogen *S*. *aureus* is a highly clonal species^16^, in which intraspecific variation is due to a large extent to the presence of mobile genetic elements like pathogenicity islands and prophages^17^. Indeed, most strains of this pathogen carry one to four prophages in their chromosome^18^. In some cases, these prophages carry accessory genes involved in virulence, such as the Panton-Valentine leukocidin, exfoliative toxin, enterotoxin A or staphylokinase, a phenomenon known as lysogenic conversion^8^. However, most prophages do not harbor virulence-related genes in their chromosome, which suggests that they may have other physiological roles that benefit their host. All staphylococcal phages known to date belong to the order Caudovirales and are included in families Podoviridae, Siphoviridae, and Myoviridae^17^. In addition to their role in the pathogenicity and evolution of their host, staphylophages have been widely used for the genetic manipulation of *Staphylococcus*, such as the well-studied siphoviruses ϕ11 and ϕ80α^17^. *S*. *aureus* can form biofilms on different environments, including food industry surfaces, implanted devices and biotic surfaces like human tissues^19^. This ability makes this pathogen able to withstand harsh conditions such as antimicrobial pressure. Since natural communities of *S*. *aureus* are expected to carry prophages, it would be interesting to determine whether their presence has an effect on the development of staphylococcal biofilms. However, the impact of phages on *S*. *aureus* biofilm formation has not been studied in depth. Thus far, two articles assessed the impact of virulent phages on the development of biofilms by this microbe^20,21^, while the influence of temperate phages remains to be elucidated. Also, there is no information available regarding the influence of lysogeny on the transcriptome of biofilm-forming cells. With this in mind, this study aimed to determine the impact of temperate phages ϕ11 and ϕ80α on the physiology of *S*. *aureus* cells during biofilm development. This knowledge will shed light on the interplay between prophages and their bacterial hosts in natural sessile communities; an information that is particularly relevant given the prevalence of lysogenic strains in this pathogen. Moreover, a better understanding of these interactions will be useful for designing strategies to eradicate biofilms in industrial and hospital settings.

## Results

### Effect of lysogenization with phages ϕ11 and ϕ80α on biofilm formation of *S*. *aureus* RN450

**Figure 1:**
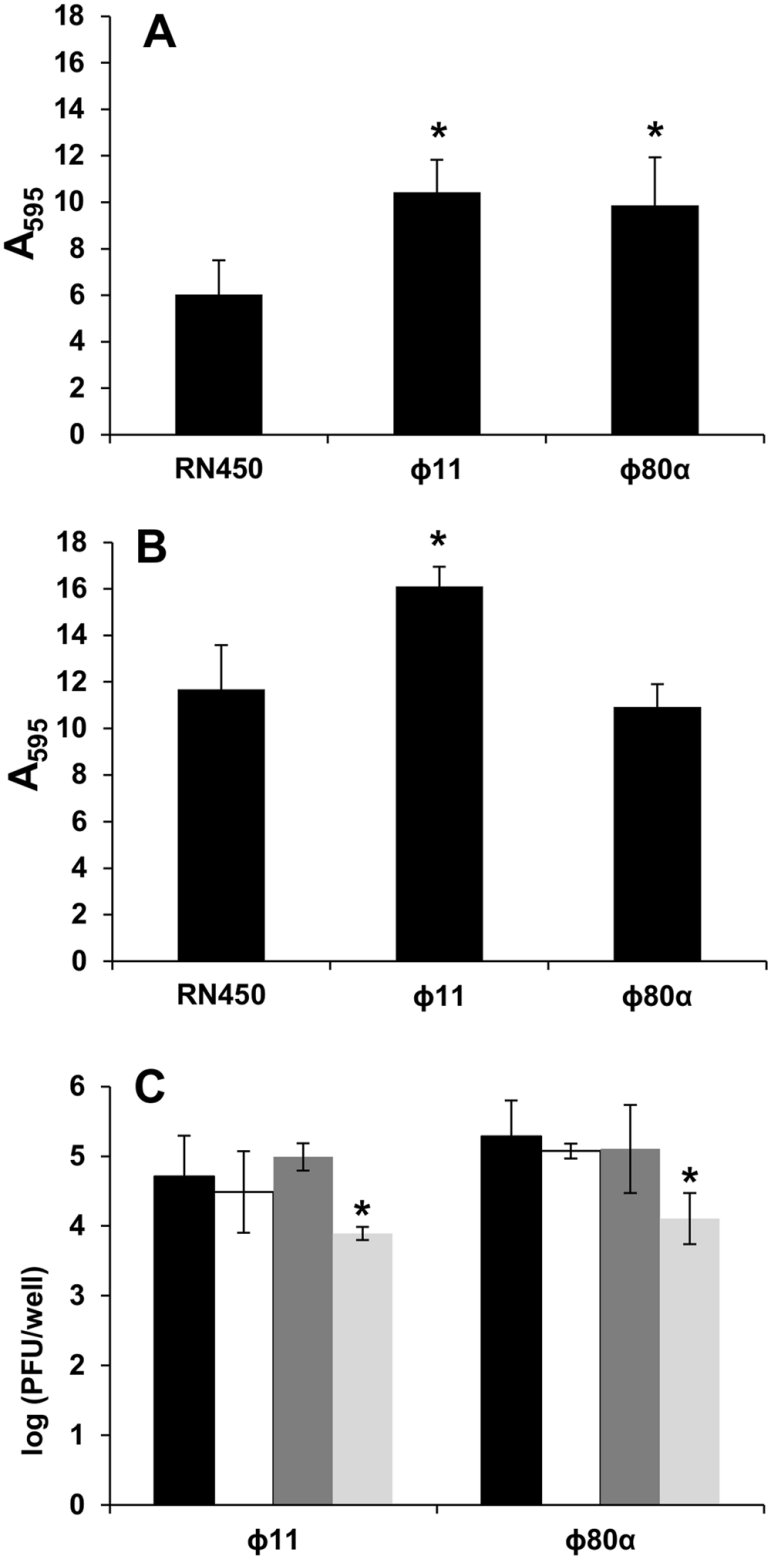
Analysis of biofilms formed by the non-lysogenic strain RN450 and the lysogenic strains RN450-ϕ11 and RN450-ϕ80α. Biofilms were formed for 5 hours (**A**) or 24 hours (**B**) and then analyzed by crystal violet staining and subsequent measurement of A_595_. (**C**) Titration of phage particles released by spontaneous induction in 5 h biofilms formed by the lysogenic strains RN450-ϕ11 and RN450-ϕ80α. Black bars, phages present as free virions in the planktonic phase; white bars, phages associated with cells present in the planktonic phase; dark gray bars, free phages present in the adhered (biofilm) phase; light gray bars, infectious centers in the adhered (biofilm) phase. The results correspond to the means and standard deviation of 3 independent biological replicates. *P-value < 0.05.

Previous studies have shown that the presence of prophages in the bacterial chromosome may have an impact on biofilm formation. Here, we sought to determine if lysogenization with the well-characterized phages ϕ11 and ϕ80α affects the development of biofilms by *S*. *aureus*. To make sure that all strains used for this study had the same genetic background, the two phages were isolated and used to lysogenize strain RN450 to obtain strains RN450-ϕ11 and RN450-ϕ80α. Then, biofilms of the three strains (lysogens and non-lysogenic strain) were allowed to develop for 5 and 24 hours. At the earlier time point, both lysogenic strains showed an increase in adhered biomass of 78% and 68% for strains carrying phages ϕ11 and ϕ80α, respectively (Fig. 1A). However, the results obtained for 24-hour biofilms were different. Thus, the strain carrying ϕ11 prophage still displayed 39% greater biofilm formation than the non-lysogenic strain, whereas the ϕ80α lysogen showed no significant (P-value = 0.48) difference compared to strain RN450 (Fig. 1B).

### Spontaneous release of phage particles in *S*. *aureus* RN450 biofilms

Once established that the presence of the two prophages affects biofilm formation in *S*. *aureus* RN450, we assessed whether there was spontaneous induction of the lytic cycle in the biofilm after 5 hours of development. It must be noted that preliminary data had shown that the maximum phage titer in the biofilm of these two lysogenic strains is precisely at 5 hours of development, later decreasing slightly at 8 h and even further at 24 h (Fig. S1). For both lysogenic strains, RN450-ϕ11 and RN450-ϕ80α, phage particles were found in both the planktonic phase and the surface-attached biomass of 5-hour biofilms (Fig. 1C). The total titer for both phages was approximately 10^5^ PFUs per well. Also, virions were isolated not only free in the supernatant or the extracellular matrix, but also inside *S*. *aureus* cells (infectious centers) (Fig. 1C). The distribution of phage particles in the different fractions was the same for both prophages. Thus, the number of PFUs in the planktonic phase (both free and cell-associated) and in the extracellular matrix of the biofilm was very similar (~10^5^ PFU/well), and higher than those found inside biofilm cells (~10^4^ PFU/well).

### Transcriptional analysis of bacterial genes in the presence of prophages

**Figure 2:**
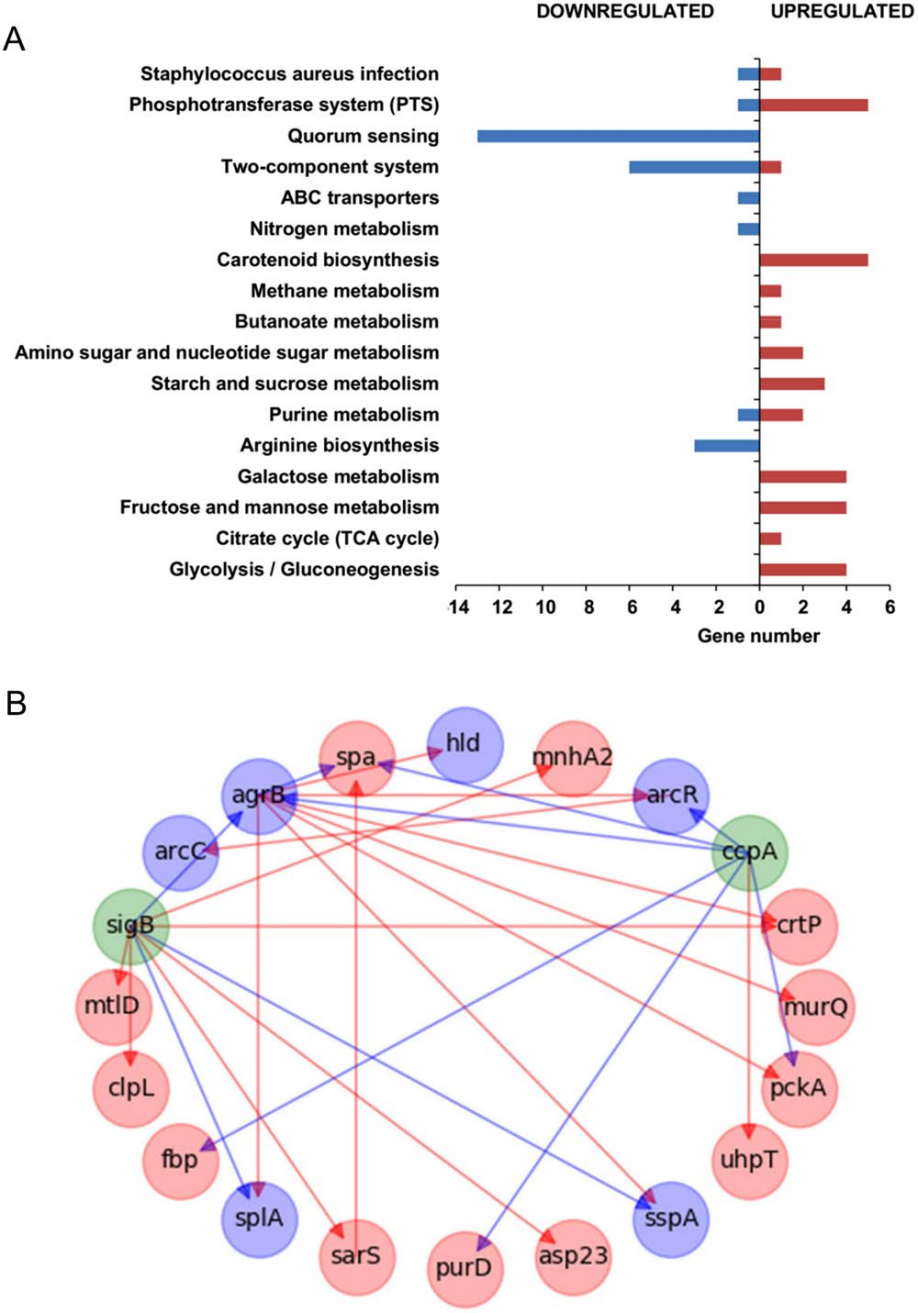
Effects of lysogenization on the transcriptome of *S*. *aureus* RN450. (**A**) KEGG pathway analysis of genes up- (red) and down-regulated (blue) in both lysogenic strains compared to the non-lysogenic strain. Bars indicate the number of genes belonging to those pathways that changed in both lysogenic strains. (**B**) Overlap of genes dysregulated in both lysogens with the SigB and CcpA regulons. Green nodes represent regulators SigB and CcpA, which did not show changes in gene expression in the RNA-seq analysis. Red and blue nodes represent up- and down-regulated genes identified by RNA-seq analysis, respectively. Red and blue arrows represent a positive and negative effect on the expression of another gene, respectively.

With the aim of studying more in-depth the impact of prophages ϕ11 and ϕ80α on bacterial physiology, we performed genome-wide transcriptional analysis of biofilms formed by RN450, RN450-ϕ11 and RN450-ϕ80α by RNA-seq. In samples corresponding to strains RN450 and RN450-ϕ11, 100% of sequences aligned with *S*. *aureus* NCTC8325. In the case of strain RN450-ϕ80α, 99% of sequences aligned with the reference *S*. *aureus* strain and 1% aligned with the phage ϕ80α genome. These results are consistent with the fact that bacteriophage ϕ11 is part of the NCTC8325 reference genome, whereas ϕ80α is not. Differential gene expression between samples from the lysogenic strains and the phage-free strain RN450 was also determined. The data obtained from this analysis revealed significant changes in the bacterial transcriptome as a result of lysogenization. It must be noted that prophage genes were excluded from this analysis and that only genes displaying changes in expression ≥2 with an adjusted P-value ≤ 0.05 were selected. Considering these parameters, the lysogenic strain RN450-ϕ11 showed upregulation of 68 genes and downregulation of 38 genes compared to the non-lysogenic strain (Table S1). Similarly, RN450-ϕ80α exhibited increased expression of 176 genes and decreased expression of 111 genes compared to strain RN450 (Table S2). There was a considerable overlap between the transcriptional changes observed in both lysogenic strains, with the results obtained for RN450-ϕ80α practically including all genes dysregulated in RN450-ϕ11 (Table 1). The only exceptions included a gene encoding a hypothetical phage protein, as well as genes *lacA* and *lacB*, all of which were slightly downregulated in the ϕ11 lysogen but not in the ϕ80α lysogen (Table S1). On the other hand, there were many genes dysregulated in the ϕ80α–harboring strain that did not change in the ϕ11 lysogen (Table S2). The genes dysregulated in both lysogenic strains were assigned to different pathways according to the KEGG database (Fig. 2A). Amongst the upregulated genes, there were a significant number of genes related to different metabolic pathways. Notably, many genes were involved in pathways related to carbohydrate metabolism, phosphotransferase systems, energy metabolism and carotenoid biosynthesis. Most of the genes that showed lower expression in the lysogenic strains were related to quorum-sensing signaling pathways and two-component regulatory systems.

**Table 1:** Genes identified by RNA-seq analysis that were dysregulated in both lysogenic strains (RN450-ϕ11 and RN450-ϕ80α) compared to prophage-free strain RN450 and overlaps with sigma B- and CcpA-regulated genes.

Gene^a^	Gene name	ϕ11^b^	ϕ80α^b^	SigB regulon^c^	CcpA regulon^c^
SAOUHSC_00061		2.35	4.97	Up	
SAOUHSC_00069*	*spa*	3.70	5.40		Up
SAOUHSC_00070	*sarS*	3.26	4.73	Up	
SAOUHSC_00156		2.47	3.18		Up
SAOUHSC_00157	*murQ*	3.13	4.18		Up
SAOUHSC_00158		3.39	4.35		Up
SAOUHSC_00160		2.31	3.46		Up
SAOUHSC_00183	*uhpT*	3.72	7.31		Up
SAOUHSC_00196	*fadB*	2.82	4.12		Up
SAOUHSC_00257		0.48	0.28		
SAOUHSC_00291		2.32	2.18		Up
SAOUHSC_00317		2.81	3.78		Up
SAOUHSC_00356		3.96	11.49	Up	
SAOUHSC_00358		4.67	12.22	Up	
SAOUHSC_00401		0.33	0.16		
SAOUHSC_00619		5.66	21.10	Up	
SAOUHSC_00624		2.72	7.23	Up	
SAOUHSC_00625	*mnhA2*	2.10	4.29	Up	
SAOUHSC_00626	*mnhB2*	2.14	3.70	Up	
SAOUHSC_00627	*mnhC2*	2.13	3.97	Up	
SAOUHSC_00628	*mnhD2*	2.14	3.75	Up	
SAOUHSC_00629	*mnhE2*	2.08	3.58	Up	
SAOUHSC_00630		2.05	3.51		
SAOUHSC_00632	*mnhG2*	2.04	3.42	Up	
SAOUHSC_00655		2.07	2.76		Up
SAOUHSC_00831		3.48	9.45	Up	
SAOUHSC_00845		8.90	31.38	Up	
SAOUHSC_00846		2.97	8.59		
SAOUHSC_00988*	*sspA*	0.49	0.32	Down	
SAOUHSC_01017	*purH*	2.17	2.57		
SAOUHSC_01018	*purD*	2.00	2.43		
SAOUHSC_01135		0.40	0.06		
SAOUHSC_01136		0.44	0.06		
SAOUHSC_01318		2.18	2.48		Up
SAOUHSC_01513		0.49	0.26		
SAOUHSC_01601		2.70	4.10		
SAOUHSC_01602		2.67	3.98		
SAOUHSC_01603		2.21	2.89		
SAOUHSC_01729		7.55	18.24	Up	
SAOUHSC_01730		6.31	19.71	Up	
SAOUHSC_01794		3.77	5.80		
SAOUHSC_01910	*pckA*	3.14	4.92		Up
SAOUHSC_01918		2.01	2.89		
SAOUHSC_01920		2.35	3.23		
SAOUHSC_01921		2.21	2.74		
SAOUHSC_01935	*splF*	0.46	0.47		
SAOUHSC_01939	*splC*	0.47	0.47	Down	
SAOUHSC_01941	*splB*	0.40	0.32	Down	
SAOUHSC_01942	*splA*	0.36	0.26	Down	
SAOUHSC_01945		0.46	0.41		
SAOUHSC_01949		0.48	0.36		
SAOUHSC_01950		0.46	0.32		
SAOUHSC_01951		0.48	0.31		
SAOUHSC_01952		0.48	0.30		
SAOUHSC_02137	*sdcS*	2.15	3.17		Up
SAOUHSC_02163		0.19	0.08		
SAOUHSC_02240		0.31	0.11		
SAOUHSC_02260*	*hld*	0.40	0.04		
SAOUHSC_02261*	*agrB*	0.50	0.17		
SAOUHSC_02265		0.50	0.20		
SAOUHSC_02266		0.45	0.24		
SAOUHSC_02387		3.30	11.15	Up	
SAOUHSC_02400		3.31	8.23		
SAOUHSC_02401		3.63	9.77	Up	
SAOUHSC_02402		4.08	11.06	Up	
SAOUHSC_02403	*mtlD*	3.27	8.74	Up	
SAOUHSC_02425		2.39	3.26		Up
SAOUHSC_02441	*asp23*	9.56	28.18	Up	
SAOUHSC_02442		7.60	27.27	Up	
SAOUHSC_02443		8.17	29.88	Up	
SAOUHSC_02444		4.99	19.92	Up	
SAOUHSC_02451		0.44	0.49		
SAOUHSC_02452	*lacD*	0.38	0.45		
SAOUHSC_02453	*lacC*	0.39	0.49		
SAOUHSC_02597		2.12	2.90		Up
SAOUHSC_02620		0.45	0.24	Down	
SAOUHSC_02729		2.20	3.14		Up
SAOUHSC_02771		2.96	5.46	Up	
SAOUHSC_02772		2.32	6.49	Up	
SAOUHSC_02812		3.00	7.98	Up	
SAOUHSC_02815		2.63	4.38		Up
SAOUHSC_02822	*fbp*	2.31	3.17		Up
SAOUHSC_02848	*glcB*	2.07	2.90		
SAOUHSC_02862	*clpL*	5.24	16.79	Up	
SAOUHSC_02877	*crtN*	2.14	5.93	Up	
SAOUHSC_02879	*crtM*	3.46	10.17	Up	
SAOUHSC_02880	*crtQ*	3.88	13.11	Up	
SAOUHSC_02881*	*crtP*	3.08	10.10	Up	
SAOUHSC_02882	*crtO*	2.93	8.93	Up	
SAOUHSC_02905		0.35	0.15		
SAOUHSC_02906		0.31	0.14		
SAOUHSC_02907		0.35	0.14		
SAOUHSC_02908		2.49	5.89	Up	
SAOUHSC_02964	*arcR*	0.42	0.24		Down
SAOUHSC_02965	*arcC2*	0.34	0.24		Down
SAOUHSC_02967		0.33	0.22		Down
SAOUHSC_02968	*argF*	0.38	0.22		Down
SAOUHSC_02969	*arcA*	0.34	0.22		Down
SAOUHSC_02970		0.48	0.19		
SAOUHSC_02971		0.45	0.30	Down	
SAOUHSC_03028		2.34	6.42	Up	
SAOUHSC_03032		2.09	4.46	Up	
SAOUHSC_A00354		5.17	13.93		

^a^Genes marked with an asterisk (*) were confirmed by RT-qPCR; the reference gene used to calculate the fold-change was *rplD*.

^b^Changes in gene expression below 1 (<1) indicate downregulation in the lysogen, whereas changes above 1 (>1) indicate upregulation in the lysogenic strain.

^c^Genes under SigB and CcpA control.

Interestingly, several genes that exhibited expression changes in the presence of the two prophages are potentially involved in biofilm formation (Table 1). For instance, the gene coding for the adhesion protein A (*spa*) was upregulated in both lysogens, which is consistent with the literature^22^. In contrast, several genes encoding dispersion factors, such as proteases (*sspA*, *splF*, *splC*, *splB*, *splA*) or surfactants (*hld*), were downregulated in both strains. Additionally, upregulation of other proteins involved in adhesion was observed in RN450-ϕ80α, such as *fnbA* and *clfA*. However, in strain RN450-ϕ80α there was also a slight downregulation of *icaB* and *icaD* (Table S2). These two genes are involved in the production of polysaccharide intercellular adhesin (PIA), which is a major component in the extracellular matrix of *S*. *aureus* biofilms.

Some genes that encode proteins with a regulatory function, such as *agrB*, *arcR*, *sarS* or *clpL*, were dysregulated in both lysogenic strains. For instance, *agrB*, which encodes a protein that participates in quorum-sensing signaling, was downregulated. This would explain, at least to some extent, the general downregulation of genes under quorum-sensing control, including proteases and *hld*. In RN450-ϕ80α there was also a lesser expression of hemolysin-encoding genes *hlgB*, *hlgC* and *hly* (Table S2), which are also under the control of Agr. ArcR is a regulator of the *arcABDC* operon, which is necessary for using arginine as a source of energy under anaerobic conditions^23^, and was found to be downregulated in both lysogens. In contrast to *agrB* and *arcR*, the SarS-encoding gene was upregulated in both lysogenic strains. In strain RN450-ϕ80α, another SarA homolog, SarT, was also induced. Finally, *clpL*, encoding an Hsp100/Clp ATPase belonging to a family of chaperones that can form a proteolytic complex with the intracellular protease ClpP, was also upregulated in both lysogenic strains^24^.

As mentioned previously, both prophages led to changes in the expression of multiple genes that participate in different metabolic pathways. For example, genes involved in nucleotide metabolism (*purH* and *purD*) and fatty acids metabolism (*fadB*) were upregulated in both lysogenic strains. Notably, there were a considerable number of genes related to carbohydrate metabolism with altered expression in the presence of the prophages. For instance, there were genes involved in glycolysis/gluconeogenesis, such as *fbp* (fructose-1,6-bisphosphatase) and *pckA* (phosphoenolpyruvate carboxykinase), as well as genes *lacC* and *lacD*, which belong to the tagatose-6-phosphate pathway. Other genes related to carbohydrate metabolism were *mtlD* (mannitol-1-phosphate 5-dehydrogenase) and *glcB* (a glucoside-specific PTS system). Additionally, several genes involved in transport were upregulated in both lysogenic strains. These genes included *mnhG2*, *mnhA2*, *mnhE2*, *mnhB2*, *mnhD2* and *sdcS* (sodium dependent dicarboxylate transporter) and the gene coding for the membrane hexose phosphate transporter protein UhpT.

Some of the genes mentioned above are known to be under the control of the catabolite control protein A (CcpA), even though the gene coding for this regulator was not identified in the transcriptional analysis (fold changes for RN450-ϕ80α and RN450-ϕ11 were 1.45 ± 0.11and 1.21 ± 0.08, respectively). Indeed, comparison of the genes dysregulated in both lysogenic strains with the information available on the CcpA regulon in the RegPrecise database (http://regprecise.lbl.gov/RegPrecise/regulog.jsp?regulog_id=662) led to the identification of 23 genes (Table 1).

Further examination of the genes that changed in both lysogenic strains revealed a significant overlap (42 genes) with the SigB regulon (Table 1), indicating a potential activation of the alternative sigma factor in the presence of the two prophages^25^. It must be noted that genes *rsbV*, *rsbW*, *sigB* and *rsbX* were not dysregulated, with respective fold-changes in RN450-ϕ11 of 1.04 ± 0.09, 0.97 ± 0.07, 0.85 ± 0.14 and 1.04 ± 0.02. Similar results were observed in RN450-ϕ80α, with fold-change values of 1.21 ± 0.02, 1.14 ± 0.02, 1.09 ± 0.06 and 1.03 ± 0.01for genes *rsbV*, *rsbW*, *sigB* and *rsbX*, respectively. Some of the most characteristic genes in the SigB regulon include *asp23*, encoding the alkaline shock protein, and the operon responsible for the biosynthesis of staphyloxanthin, the characteristic yellow pigment of *S*. *aureus*^25^. All of these genes were upregulated in the two lysogenic strains, although this increase was more pronounced in RN450-ϕ80α than in RN450-ϕ11.

### Transcriptional analysis of bacteriophage gene expression

**Figure 3:**
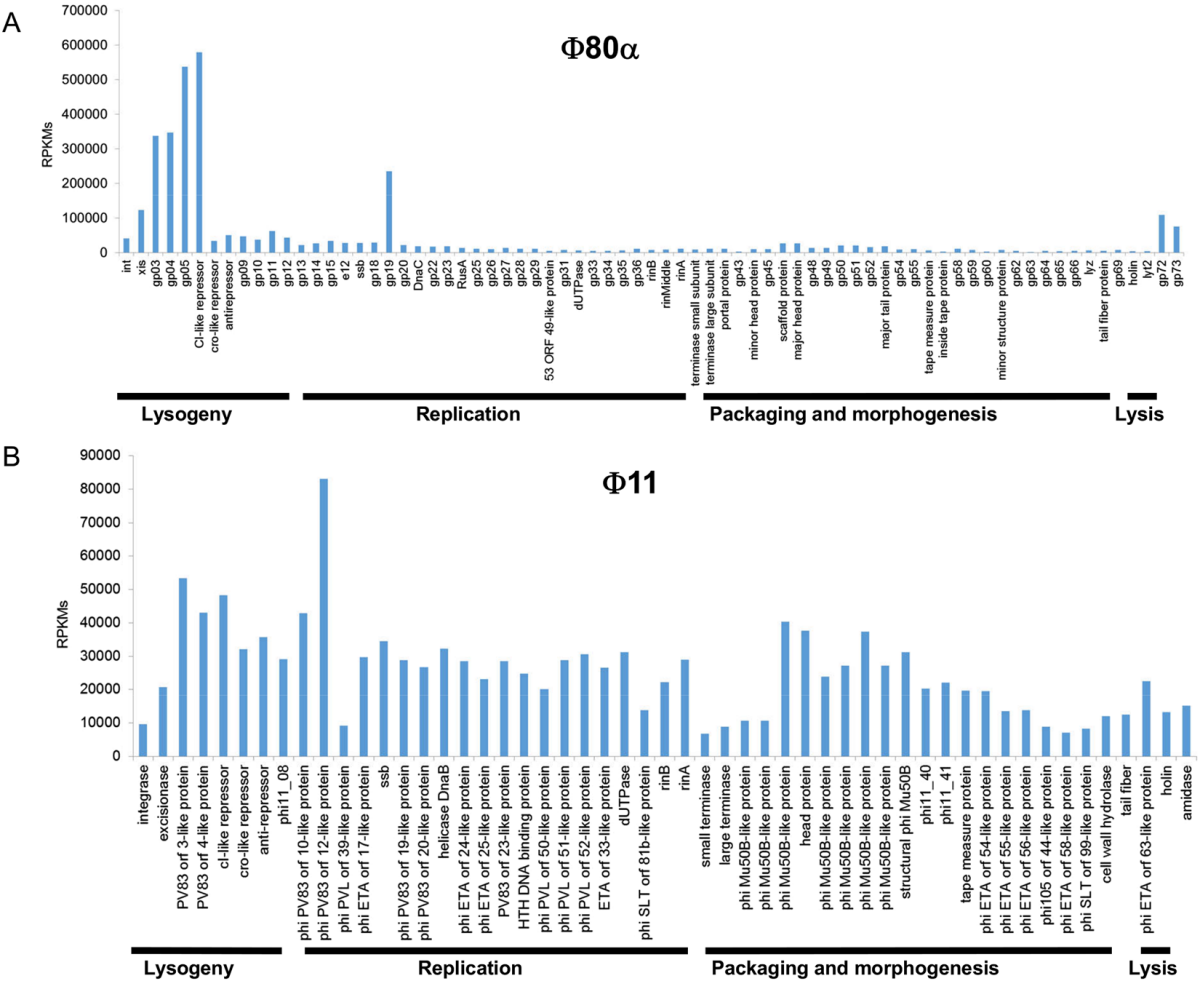
Transcriptome of prophages ϕ80α and ϕ11. Normalized mean reads per kilobase million (RPKM) values corresponding to the different open reading frames (ORFs) from the genome of phages ϕ80α (**A**) and ϕ11 (**B**).

In addition to studying transcriptional changes in bacterial genes, RNAseq data also provided information regarding the expression of phage genes in the samples corresponding to the lysogenic strains. In the case of RN450-ϕ80α, although there was some level of expression of all genes throughout the genome, the highest transcription levels were found in genes of the lysogeny module (Fig. 3A). Thus, the highest expression was displayed by genes gp05 and the gene encoding the CI protein, with RPKM values between 500,000 and 600,000. Next in expression levels were gp03 and gp04, with RPKM values of 300,000. Unexpectedly, a gene in the replication module, gp19, exhibited a high expression, with RPKM values of approximately 200,000. The excisionase-encoding gene *xis* and hypothetical proteins gp72 and gp73 had RPKM values of about 100,000. All other ORFs showed RPKM values below 100,000. In the case of *xis*, it must be noted that the transcription levels quantified here may reflect reads of the transcriptional unit encoding gp03 which is encoded in the complementary strand. In order to determine if this is the case, strand specific RNA-seq would have to be performed. The transcriptional profile of phage ϕ11 was quite different. At first glance, it can be observed that expression was more evenly distributed along the genome and no specific region was overrepresented (Fig. 3B). The gene with the highest level of expression, with RPKM values of 80,000, was phi11_10, which belongs to the replication module.

### Effect of mutations in different phage ϕ80α genes on biofilm formation, phage release and staphyloxanthin production

**Figure 4:**
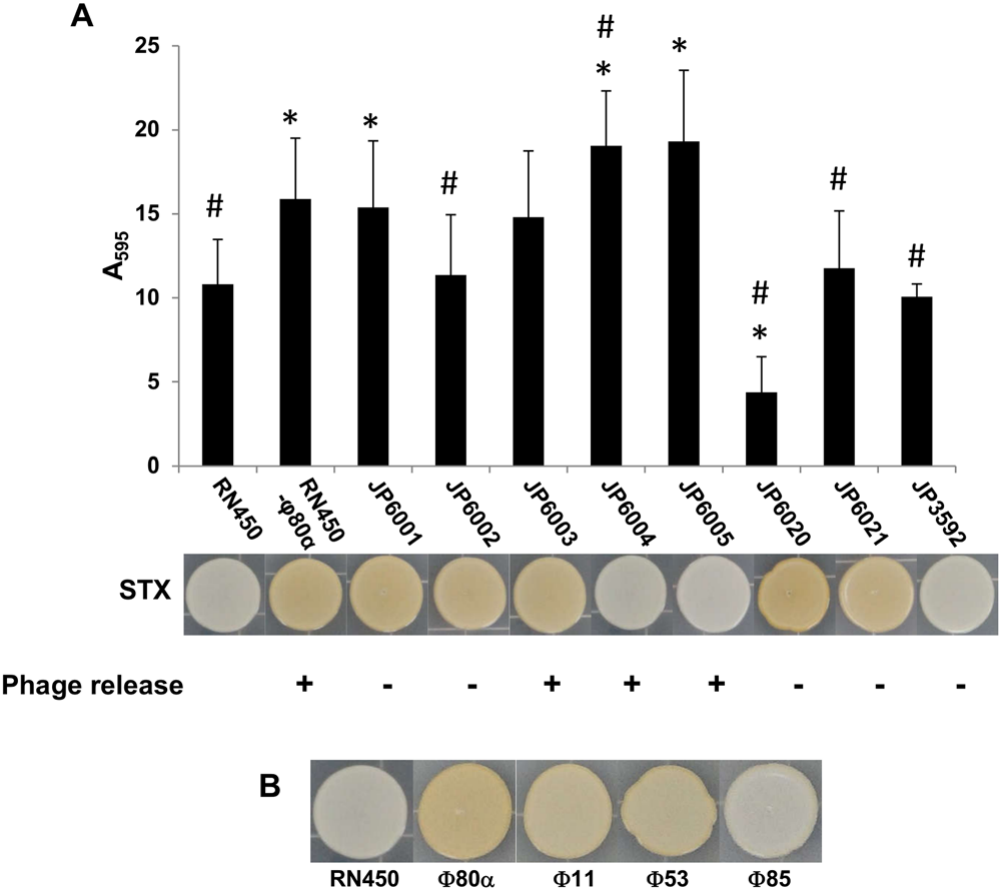
Involvement of phage genes in biofilm formation and staphyloxanthin production in lysogenic strains. (**A**) Biofilm formation (black bars), staphyloxanthin production and spontaneous release of virions of mutants derived from the lysogenic strain RN450-ϕ80α after 5 hours of incubation at 37 °C. Values represent the average and standard deviation of 4 independent experiments. *Differences compared to RN450 were statistically significant (p-values < 0.05); ^#^Differences compared to RN450-ϕ80α were statistically significant (p-values < 0.05). (**B**) Staphyloxanthin (STX) production of *S*. *aureus* RN450 and its derived lysogenic strains.

In an attempt to characterize the mechanisms involved in the phenotypic and physiological changes observed in the lysogenic strains, several mutant strains derived from RN450-ϕ80α were analyzed (Table 2). Some of these strains had deletions affecting genes involved in prophage integration into or excision from the bacterial chromosome (JP6001 and JP6002), genes involved in phage DNA replication (JP6020 and JP6021) or genes of unknown function located in the lysogeny module (JP6003, JP6004 and JP6005). Also, there was one strain with a point mutation in the CI protein that made it insensitive to the SOS response (JP3592), thereby abrogating induction of the lytic cycle. First, prophage release upon mitomycin C induction was examined. We observed that strains RN450-ϕ80α, JP6003, JP6004 and JP6005 exhibited similar levels of phage release, with respective phage titers of 7.62 ± 0.60, 8.08 ± 0.45, 7.7 ± 0.74 and 7.85 ± 0.73 log (PFU/ml). In contrast, the mutant strains JP6001, JP6002, JP6020, JP6021 and JP3592 did not exhibit detectable levels of released phage particles (Fig. 4A).

**Table 2:** Bacterial strains used in this study.

*S*. *aureus* strains	Description	Reference
RN450	NCTC8325 strain curated of prophages ϕ11, ϕ12 and ϕ13	^55^
RN451	RN450 lysogenized with prophage ϕ11	^55^
RN10359	RN450 lysogenized with prophage ϕ80α	^56^
RN450-ϕ11	RN450 lysogenized with prophage ϕ11	This study
RN450-ϕ80α	RN450 lysogenized with prophage ϕ80α	This study
RN450-ϕ53	RN450 lysogenized with prophage ϕ53	This study
RN450-ϕ85	RN450 lysogenized with prophage ϕ85	This study
JP6001	ϕ80α Δ *int* (*gp01*)	This study
JP6002	ϕ80α Δ *xis* (*gp02*)	This study
JP6003	ϕ80α Δ *gp03*	This study
JP6004	ϕ80α Δ *gp04*	This study
JP6005	ϕ80α Δ *gp05*	This study
JP6020	ϕ80α Δ *gp20*	This study
JP6021	ϕ80α Δ *gp21*	This study
JP3592	ϕ80α *cI* G130E (phage mutant non-responsive to SOS activation)	^6^

Next, we assessed whether these mutations had an impact on the biofilm formation ability of the lysogenic strain. The results of these assays showed that deletion of genes *xis*, gp20 or gp21 or a point mutation in the *cI* gene led to a lesser biofilm formation compared to the parent lysogenic strain RN450-ϕ80α (Fig. 4A). Indeed, the mutant in gp21 exhibited an even lower biofilm-forming ability than the nonlysogen RN450. In contrast, strains with deletion mutations in gp04 and gp05 showed greater biofilm development than RN450; in fact, JP6004 formed even more biofilm than RN450-ϕ80α (Fig. 4A). Strain JP6003 showed no significant difference in biofilm formation compared to RN450 or RN450-ϕ80α (Fig. 4A).

Another phenotype of interest was staphyloxanthin production, which was visualized after growth of bacterial colonies for 48 hours on TSA plates. As mentioned above, strains carrying ϕ11 and ϕ80α as prophages showed an increased expression of genes involved in the biosynthesis of the carotenoid pigment staphyloxanthin. As predicted by transcriptional analysis, colonies of the lysogenic strain RN450-ϕ80α displayed a more intense yellow/orange color than those of RN450 (Fig. 4A). Regarding the effect of mutations in phage genes, results showed that deletion of genes gp04 and gp05 as well as a point mutation in the adjacent gp06, encoding protein CI, resulted in a lesser carotenoid production. In contrast, genes involved in integration/excision of the prophage from the chromosome (*int* and *xis*) or genes involved in phage replication (gp20 and gp21) had no impact whatsoever on staphyloxanthin production (Fig. 4A). We also compared staphyloxanthin production in lysogenic strains harboring prophages ϕ80α, ϕ11, ϕ53 and ϕ85 and observed that the most intense color was displayed by the ϕ80α lysogenic strain, followed by ϕ11 and ϕ53, which were quite similar to each other, and ϕ85, which exhibited a color akin to that of the non-lysogen RN450 (Fig. 4B). These results were interesting considering the similarity amongst the CI proteins of the four phages analyzed with the BLASTP program^26^. Thus, the identity between CI proteins of ϕ11 and ϕ53 was about 99%, whereas identity between ϕ11 or ϕ53 with ϕ80α CI protein was 76 and 77%, respectively. The CI protein from phage ϕ85 was the least similar to any of the other three proteins with 23, 30 and 21% identity with CI proteins from phages ϕ11, ϕ80α and ϕ53, respectively.

## Discussion

Prophages play an important role in the evolution and fate of microbial communities^1,2,7^. Embedded in bacterial genomes, these viral entities can enhance fitness of their host bacteria or, conversely, make them more vulnerable to certain environmental challenges. Examples of the former include lysogenic conversion, in which the phage genetic material encodes virulence factors, or enhanced biofilm development as a result of lysogenization. However, there are instances in which carrying a prophage may be lethal in the presence of a triggering factor of the lytic cycle. This has been observed in *E*. *faecalis* biofilm dispersion of lysogenic strains, and the heightened susceptibility of *S*. *aureus* lysogens to the hydrogen peroxide produced by *S*. *pneumoniae*^6,12^. Until now, the impact of prophages during biofilm development had not been examined in depth in *S*. *aureus*. Within this context, this study aimed to determine how two well-known temperate phages, ϕ80α and ϕ11, altered the transcriptome of their host during biofilm formation.

The preliminary studies performed prior to the transcriptional analysis showed that both lysogenic strains exhibited greater biomass accumulation in earlier stages of biofilm development. However, biomass of mature biofilms only increased in the ϕ11 lysogen. This would suggest that the mechanism(s) responsible for biofilm increase in the two lysogenic strains may be different, at least in late biofilm developmental stages. Indeed, it appears that for the ϕ80α lysogen, biofilm formation occurs at a faster rate but ultimately leads to a similar amount of total adhered biomass. Spontaneous release of phage particles was observed for both lysogens in the planktonic phase and the biofilm. Interestingly, there were fewer virions inside biofilm cells than free in the extracellular matrix. This suggests that spontaneous induction of the lytic cycle has slowed down in sessile cells at this stage of biofilm formation. Indeed, such hypothesis was re-inforced by an additional experiment showing that the maximum phage titer in the biofilm was found at 5 hours of development (Fig. S1). Similarly, Resch *et al*.^27^ observed that maximum spontaneous phage release in the lysogenic strains *S*. *aureus* Sa113 and *S*. *aureus* Newman peaked before 8 hours of growth both in biofilms and in planktonic cultures. Also, these results hint that, upon lysis of the bacterial cells, the phage particles may accumulate in the matrix and, together with the released cell contents, contribute to the total biomass of the biofilm. In fact, spontaneous induction of the lytic cycle had been previously linked to increased biofilm formation in some bacteria^13^. Moreover, this “phage network” may act as a defense mechanism against competing susceptible strains as well as enhance genetic transfer within the biofilm. Indeed, Haaber *et al*.^28^ already demonstrated that prophages can promote transfer of antibiotic resistance markers from neighboring cells into prophage-carrying bacteria. Taken together, all these data indicate that prophages may contribute to the increase in horizontal gene transfer observed in biofilms^29,30^.

Transcriptional analysis of prophage ϕ80α genes showed a higher level of expression of genes in the lysogeny module. Interestingly, phage Tuc2009 exhibited a similar expression pattern during lysogeny of *Lactococcus lactis* UC509.9^31^. Indeed, the genes displaying the highest level of expression were those in the lysogeny module, especially the lytic cycle repressor, and one gene in the early replication module. In contrast to the results presented here, previous transcriptomic analysis of phage ϕ80α in *S*. *aureus* had shown expression distributed more evenly across the genome during lysogeny^32^. It is very likely that the discrepancies in the expression profile between the two studies are due to the fact that the lysogen cells were in two different growth states. Thus, while Quiles-Puchalt *et al*.^32^ examined transcription in a liquid culture during early exponential phase, the analysis described here corresponds to biofilm cells after 5 hours of development, which corresponds more accurately to late exponential phase. The results obtained for ϕ11, however, revealed a more homogeneous expression of genes throughout the phage genome, a profile that seems to correspond to a population in different stages of the lysogenic/lytic cycle. Interestingly, the gene displaying the highest level of transcription was located in the replication module. A higher gene expression in the gene replication and gene regulation module was also found in the transcriptomic analysis of a *Clostridium difficile* lysogenic strain carrying prophage phi-027^33^.

Transcriptional profiling of biofilms formed by the two lysogenic strains and the prophage-free strain revealed major changes associated with the presence of the prophages. A closer look at the results from these analyses indicates that strains harboring temperate phages exhibit differences in genes affecting metabolism, virulence, biofilm formation or production of the protective pigment staphyloxanthin. From a regulatory perspective, many of the genes with differential expression in lysogenic strains belong to the SigB, CcpA and Agr regulons (Table 1 and Fig. 2B). However, the exact regulatory cascade between these systems in response to the phages remains unknown. Similarly, it is difficult to find a direct connection between the changes in gene expression and the biofilm phenotypes of the two strains. Thus, some of the genes dysregulated in both lysogens could explain an increased biofilm formation, although no hint was found regarding the lack of biofilm increase at 24 hours of development in RN450-ϕ80α. For example, extracellular proteases, which are known to reduce biofilm formation in *S*. *aureus*^34^, were downregulated in the lysogens. This may be related to the lesser expression of genes belonging to the Agr quorum sensing system, which is known to participate in biofilm dispersion in *S*. *aureus*^35,36^. Also, there is increased expression of genes coding for proteins involved in adhesion and in production of extracellular polysaccharide. Other genes that may alter biofilm formation in the presence of the prophages are those encoding regulatory proteins SarS, SarT or ClpL. SarS is one of several SarA-like regulators that participate in the complex network controlling virulence-gene expression in *S*. *aureus*^37^. In particular, SarS is a positive regulator of *spa*, the gene that codes for staphylococcal protein A, which is involved in virulence-related traits as well as adherence^22^. Interestingly, SarT was previously found to induce expression of SarS^38^. ClpP, on the other hand, is one of the ATPases that form the ClpP proteolytic complex, which is known to have a pleiotropic effect on *S*. *aureus*, participating in processes like biofilm formation, virulence, antibiotic resistance and survival under stressful conditions^24,39^. However, the specific role of ClpL in *S*. *aureus* has not been well defined yet, although a study by Frees *et al*.^24^ revealed its involvement in the multiplication of the pathogen inside bovine mammary epithelial cells.

As mentioned previously, there are several dysregulated genes in prophage-carrying strains that are known to be under the control of CcpA. However, some of the identified genes exhibited changes indicating that CcpA was activated in the lysogens, whereas other dysregulated genes appeared to indicate the opposite. For example, CcpA activation could explain the higher expression of *uhpT* and the lower expression of *arcR* and *arcABDC*^40^. In contrast, the upregulation of *spa*, *purD*, *pckA* and *fbp* would suggest inhibition of CcpA activity^40^. Interestingly, deletion of CcpA in *S*. *aureus* SA113 inhibited biofilm formation as it decreased production of PIA and accumulation of eDNA^41^. In a previous work, Seidl *et al*.^42^ had also demonstrated that gene regulation by CcpA modulated expression of antibiotic and virulence determinants in *S*. *aureus* by studying the transcriptional changes displayed by *ccpA* deletion mutants derived from strains COLn and Newman.

The sigma B regulon is typically induced under stress conditions, including heat, oxidative and antibiotic stress. One of the genes upregulated by SigB is *asp23*, which codes for the alkaline shock protein Asp23, a membrane-anchored protein thought to participate in homeostasis of the cell envelope^43^. Another effect of this response is the increased production of staphyloxanthin. This carotenoid pigment embeds itself into the cytoplasmic membrane and has been shown to promote virulence, hamper killing by neutrophils and decrease susceptibility to cationic antimicrobial peptides^44,45^. The alternative sigma factor also participates in the regulation of biofilm formation in *S*. *aureus* by leading to decreased expression of extracellular dispersion factors, like proteases^25^. Thus, activation of the SigB regulon may be to some extent related to the increased attached biomass displayed by the lysogenic strains. Moreover, biofilm formation is a recognized persistence strategy of bacterial pathogens that marks the transition from an acute infection to a chronic infection^46^. In that sense, it has been recently shown that activation of the SigB regulon and inactivation of the Agr regulon are necessary for adaptation of *Staphylococcus* during chronic infections^46^. All this information suggests that prophage carriage may be beneficial for the microbe during the infection process, a possibility that should be the focus of subsequent studies. In that sense, it must be highlighted that strain RN450 is a good model for studying this phenomenon precisely because of its inherent SigB-negative phenotype. Indeed, this strain is known to carry a mutation in the *rsbUVW*-*sigB* operon that leads to reduced activation of SigB^47^. Therefore, the phenomenon observed here could be regarded as if lysogenization of this strain compensates for the aforementioned mutation^47^. Conversely, staphyloxanthin production in SigB-positive strains may mask the potential impact of prophages on the regulation of the SigB regulon. Interestingly, the alternative sigma factor in *E*. *coli*, RpoS, was upregulated in a lysogenic strain carrying prophage ϕ24B^48^. A consequence of this was increased acid resistance due to upregulation of the glutamic acid decarboxylase (GAD) operon. Another interesting observation is that RpoS was found to modulate phage induction in *E*. *coli*^49^. There are also examples of prophages encoding alternative sigma factors in their genomes, as is the case of *Bacillus anthracis* phages, which have an effect on important processes like biofilm formation and sporulation^50^. A further connection between phages and sigma factors is that the RepR repressor from *C*. *difficile* temperate phage CD119 repressed the expression of the alternative sigma factor TcdR^51^. Altogether, these results reflect the existence of complex interactions between the CcpA and SigB regulons, and their respective connections to the agr quorum-sensing system (Fig. 2B). Further studies should decipher more in detail the physiological and molecular processes involved in this regulatory network.

Experiments carried out with mutant strains of prophage ϕ80α indicated that the mechanisms behind biofilm induction and greater staphyloxanthin production were different. Indeed, the biofilm phenotype seemed to correlate with spontaneous activation of the lytic cycle. This suggests that release of cellular components and phage particles due to cell lysis may be responsible for the enhanced accumulation of adhered biomass. By contrast, there was no apparent connection between staphyloxanthin production and phage particle release. It is worth noting, however, that an intact CI protein is necessary for both properties. This suggests that the CI protein may play a role within the bacterial regulatory networks. Interestingly, the regulator CI from phage ϕ11 was shown to inhibit aureocin A79 production in synergy with the bacterial regulatory protein AurR^52^. Nonetheless, further research is still necessary to determine how these interactions take place. Likewise, staphyloxanthin production varied in different lysogenic strains, carrying prophages ϕ80α, ϕ11, ϕ53 and ϕ85, which exhibit differences in the sequence of their respective CI proteins. However, no conclusions can be drawn without performing further experiments, such as studies involving heterologous complementation of the CI protein. Moreover, differences in the lysogeny module between these lysogenic strains are not limited to the CI protein. Therefore, the effect of other phage proteins on this phenotype should also be examined.

In conclusion, the results of this study show that lysogenization of *S*. *aureus* with two phages leads to increased biofilm formation and staphyloxanthin production, as well as transcriptional changes involving some of the major regulatory networks in this pathogen. Indeed, as discussed above, the interconnection between the SigB regulon and the Agr quorum-sensing signaling cascade plays a paramount role in modulating the alternation between the lifestyles associated to acute and chronic infections, as well as the development of sessile communities. In that sense, carrying a prophage may contribute to these bacterial adaptions, thereby exerting a positive impact on the host population. Moreover, it appears that mutations in certain phage genes abrogate this effect, being especially interesting the potential participation of the CI protein in regulating the expression of host genes. Nonetheless, further work is still needed to ascertain if this is really the case. Altogether, our data emphasizes again the dramatic effect of bacteriophages on microbial communities. Evidently, more studies are still required to understand how interactions between staphylophages and their host develop. However, what is becoming increasingly clear is that studying phage-host interactions will be paramount to gain more knowledge of bacterial population dynamics. In turn, this information will provide a more realistic view of the role that microbes play in natural environments as well as help to design new strategies to fight unwanted microorganisms.

## Methods

### Bacterial strains and growth conditions

All bacterial strains used in this study are listed in Table 2. Routine growth of *S*. *aureus* cultures was performed at 37 °C on Baird-Parker agar plates (AppliChem, Germany) or in TSB (Tryptic Soy Broth, Scharlau, Barcelona, Spain) with shaking. Staphyloxanthin production was monitored by inoculating 5 μl drops from overnight cultures on TSA plates containing 1.5% agar and 0.25% glucose. These plates were subsequently incubated for 48 hours at 37 °C.

### Biofilm formation assays

Biofilm assays were performed as previously described but with some modifications^53^. Thus, *S*. *aureus* strains were grown overnight and subsequently diluted to a cell count of 10^6^ cfu/ml in TSB supplemented with 0.25% w/v D-(+)-glucose (TSBG). 200 μl aliquots from these cell suspensions were poured into the wells of a 96-well microtiter plate (Thermo Scientific, NUNC, Madrid, Spain). Biofilms were allowed to develop for 24 hours at 37 °C, after which adhered biomass was quantified by crystal violet staining. Briefly, the supernatant was removed and the wells were washed twice with PBS (137 mM NaCl, 2.7 mM KCl, 10 mM Na_2_HPO_4_ and 2 mM KH_2_PO_4_; pH 7.4). Then, 200 μl of 0.1% (w/v) crystal violet were added to the wells and subsequently removed after 15 minutes of incubation. Excess crystal violet was washed with water and 33% (v/v) acetic acid was added to solubilize the dye attached to the well prior to quantification by measuring absorbance at 595 nm (A_595_) with a Bio-Rad Benchmark plus microplate spectrophotometer (Bio-Rad Laboratories, Hercules, CA, USA).

Titration of plaque forming units present in the planktonic phase and the biofilm was carried out according to the protocol described by González *et al*.^54^, in which phages were identified as being “free” or “cell-associated” (infectious centers) for each phase. The resulting samples were filtered (0.45 µm, VWR, Spain) to remove bacterial cells and titrated according to the double layer technique using strain *S*. *aureus* RN4220 as a host.

### RNA purification and analysis

Samples of 5 h biofilms grown at 37 °C were collected from two independent biological replicates for each strain (RN450, RN450-ϕ11 and RN450-ϕ80α) and total RNA was prepared as described elsewhere^21^. Purity and concentration of the RNA samples were checked by agarose gel electrophoresis and quantification with an Epoch microplate spectrophotometer (Biotek). 10 μg of RNA from each sample were sent to Macrogen Inc. (South Korea) for sequencing with the Illumina HiSeq2000 platform (Illumina, San Diego, CA, USA). Bioinformatic analysis was carried out at Dreamgenics (Dreamgenics, Oviedo, Spain). Quality of the RNA-seq reads was checked with FastQC. RNA-seq reads were then mapped to the *S*. *aureus* NCTC 8325, phage ϕ11 and phage ϕ80α genomes by using BowTie2. Uniquely mapped reads were kept for subsequent analysis of differential gene expression using EDGE-pro software. RNA-Seq data have been deposited in NCBI’s Gene Expression Omnibus (GEO) and can be accessed through GEO series accession number GSE111012.

Transcriptional changes for selected differentially-expressed genes identified in the RNA-seq analyses were confirmed by quantitative reverse transcription-PCR (RT-qPCR). Briefly, following RNA preparation, cDNA was obtained from 0.5 μg aliquots of the RNA samples by using iScript™ Reverse Transcription Supermix for RT-qPCR (BioRad). The resulting cDNA samples were then used as templates for RT-qPCR analysis using Power SYBR Green PCR Master Mix (Applied BioSystems). Three independent biological replicates were analyzed for each strain. Fold-changes were calculated following the Ct method and the reference gene was *rplD*.

### Statistical analyses

Data from independent biological replicates was analyzed with a two-tailed Student’s t-test. Significance was set at a P-value threshold of 0.05.

## Electronic supplementary material

Supplementary information
