# Supplementary material for: Lysogenization of Staphylococcus aureus RN450 by phages ϕ11 and ϕ80α leads to the activation of the SigB regulon
Source: Sci Rep. 2018 Aug 23;8:12662. doi: 10.1038/s41598-018-31107-z (PMC6107660; doi:10.1038/s41598-018-31107-z)
Supplement: Supplementary file 1 — Supplementary information [file 41598_2018_31107_MOESM1_ESM.pdf]

Lysogenization of *Staphylococcus aureus* RN450 by phages ϕ11 and ϕ80α leads to the activation of the SigB regulon

Running Title: Transcriptome of *Staphylococcus aureus* lysogens

Lucía Fernández[1#], Silvia González[1], Nuria Quiles-Puchalt[2], Diana Gutiérrez[1], José R. Penadés[2], Pilar García[1], Ana Rodríguez[1]

[1]Instituto de Productos Lácteos de Asturias (IPLA-CSIC). Paseo Río Linares s/n 33300 - Villaviciosa, Asturias, Spain.

[2] Institute of Infection, Immunity and Inflammation, College of Medical, Veterinary and Life Sciences, University of Glasgow, G12 8TA Glasgow, UK.

[#]Address correspondence to Dr. Lucía Fernández, lucia.fernandez@ipla.csic.es

L.F. and S.G. contributed equally to this work

Figure S1. Analysis of phage release throughout biofilm development in lysogenic strains RN450-ϕ11 and RN450-ϕ80α. Biofilms were formed for 3, 5, 8 or 24 hours by strains RN450-f80a (A) or RN450-f11 (B). Then, free phage particles in the adhered phase (biofilm) were titrated using strain *S. aureus* RN4220 as a host.

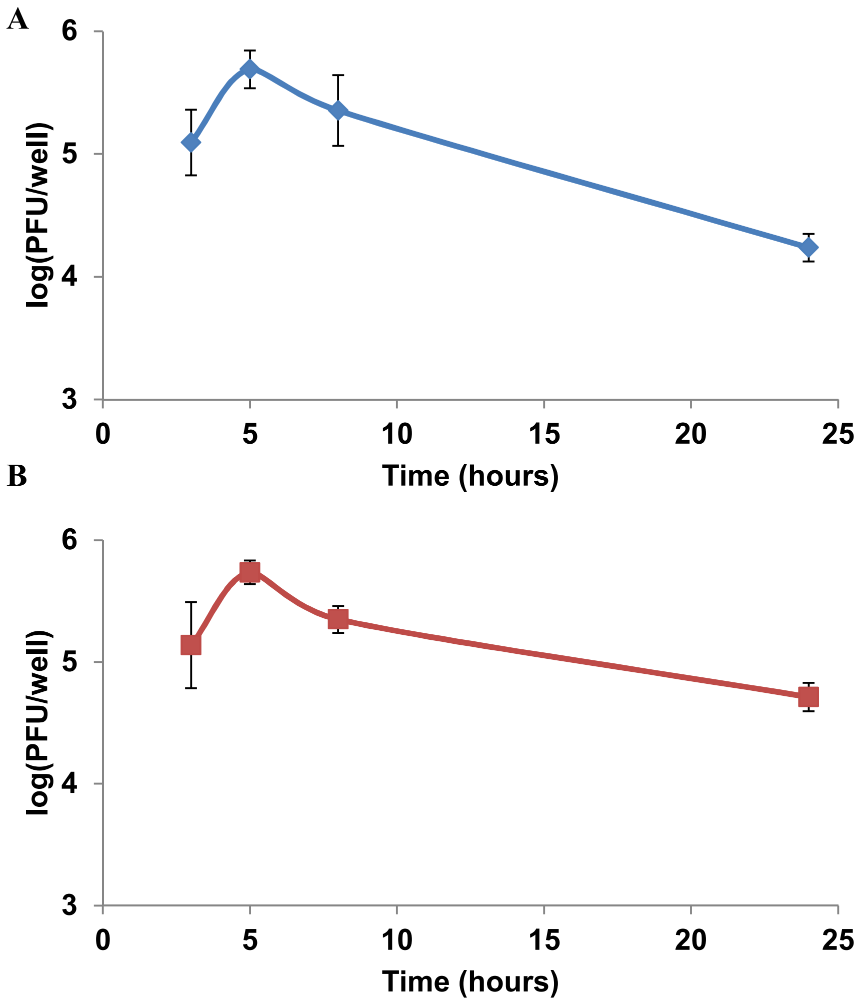

Table S1. Full list of genes dysregulated in biofilms formed by the lysogenic strain RN450-Φ11 compared to the non-lysogenic strain RN4

Gene ID*	Gene name	Gene product	Fold-change	padj
SAOUHSC_02163			-5.22	9.41E-10
SAOUHSC_02906	SAOUHSC_02906	Putative uncharacterized protein	-3.24	5.66E-08
SAOUHSC_02240			-3.21	8.61E-06
SAOUHSC_02967	SAOUHSC_02967	"Arginine/ornithine antiporter, putative"	-3.06	7.63E-09
SAOUHSC_00401	SAOUHSC_00401	Putative uncharacterized protein	-2.99	4.41E-04
SAOUHSC_02969	arcA	Arginine deiminase	-2.98	6.10E-07
SAOUHSC_02965	arcC2	Carbamate kinase 2	-2.94	9.53E-12
SAOUHSC_02907	SAOUHSC_02907	Putative uncharacterized protein	-2.89	1.29E-05
SAOUHSC_02905	SAOUHSC_02905	Putative uncharacterized protein	-2.83	5.78E-08
SAOUHSC_01942	splA	Serine protease SplA	-2.75	4.30E-04
SAOUHSC_02968	argF	Ornithine carbamoyltransferase	-2.66	2.81E-06
SAOUHSC_02452	lacD	"Tagatose 1,6-diphosphate aldolase"	-2.60	1.07E-05
SAOUHSC_02453	lacC	Tagatose-6-phosphate kinase	-2.56	2.50E-06
SAOUHSC_01941	splB	Serine protease SplB	-2.51	1.33E-02
SAOUHSC_02454	lacB	Galactose-6-phosphate isomerase subunit LacB	-2.49	4.14E-03
SAOUHSC_01135	SAOUHSC_01135	Putative uncharacterized protein	-2.49	1.91E-03
SAOUHSC_02260	hld	Delta-hemolysin	-2.48	1.54E-04
SAOUHSC_02090	SAOUHSC_02090	Conserved hypothetical phage protein	-2.45	1.21E-15
SAOUHSC_02964	arcR	HTH-type transcriptional regulator ArcR	-2.41	8.50E-06
SAOUHSC_01136	SAOUHSC_01136	Putative uncharacterized protein	-2.27	2.43E-03
SAOUHSC_02451	SAOUHSC_02451	"PTS system lactose-specific IIA component, putative"	-2.26	1.21E-02
SAOUHSC_02266	SAOUHSC_02266	Putative uncharacterized protein	-2.25	4.99E-06
SAOUHSC_02620	SAOUHSC_02620	Putative uncharacterized protein	-2.24	6.23E-06
SAOUHSC_02971	SAOUHSC_02971	"Aureolysin, putative"	-2.21	4.19E-11
SAOUHSC_01950	SAOUHSC_01950	"Flavoprotein, epiD, putative"	-2.20	1.19E-04
SAOUHSC_01945	SAOUHSC_01945	"Membrane protein, putative"	-2.17	7.81E-06
SAOUHSC_01935	splF	Serine protease SplF	-2.15	9.18E-04
SAOUHSC_01939	splC	Serine protease SplC	-2.14	1.53E-03
SAOUHSC_01949	SAOUHSC_01949	"Intracellular serine protease, putative"	-2.10	2.50E-05
SAOUHSC_00257	SAOUHSC_00257	Putative uncharacterized protein	-2.09	1.13E-05
SAOUHSC_01952	SAOUHSC_01952	"Lantibiotic epidermin biosynthesis protein EpiB, putative"	-2.07	2.41E-04
SAOUHSC_01951	SAOUHSC_01951	"Epidermin biosynthesis protein EpiC, authentic point mutatio	-2.07	1.21E-04
SAOUHSC_02970			-2.06	5.66E-04
SAOUHSC_01513	SAOUHSC_01513	Putative uncharacterized protein	-2.03	1.42E-05
SAOUHSC_00988	sspA	Glutamyl endopeptidase	-2.03	1.95E-04
SAOUHSC_02455	lacA	Galactose-6-phosphate isomerase subunit LacA	-2.01	1.42E-02
SAOUHSC_02261	agrB	Accessory gene regulator protein B	-2.00	1.08E-04
SAOUHSC_02265	SAOUHSC_02265	Accessory gene regulator protein A	-2.00	8.54E-06
SAOUHSC_01018	purD	Phosphoribosylamine--glycine ligase	2.00	1.69E-03
SAOUHSC_01918	SAOUHSC_01918	Putative uncharacterized protein	2.01	1.13E-02
SAOUHSC_00632	mnhG2	Putative antiporter subunit mnhG2	2.04	5.53E-05
SAOUHSC_00630	SAOUHSC_00630	Putative uncharacterized protein	2.05	6.38E-06
SAOUHSC_02848	glcB	PTS system glucoside-specific EIICBA component	2.07	1.21E-04
SAOUHSC_00655	SAOUHSC_00655	Putative uncharacterized protein	2.07	4.88E-02
SAOUHSC_00629	mnhE2	Putative antiporter subunit mnhE2	2.08	1.37E-05
SAOUHSC_03032	SAOUHSC_03032	Putative uncharacterized protein	2.09	4.16E-03
SAOUHSC_00625	mnhA2	Putative antiporter subunit mnhA2	2.10	2.46E-08
SAOUHSC_02597	SAOUHSC_02597	"PTS system component, putative"	2.12	1.07E-02
SAOUHSC_00627	mnhC2	Putative antiporter subunit mnhC2	2.13	2.68E-04
SAOUHSC_00626	mnhB2	Putative antiporter subunit mnhB2	2.14	7.06E-05
SAOUHSC_00628	mnhD2	Putative antiporter subunit mnhD2	2.14	5.08E-08
SAOUHSC_02877	crtN	Dehydrosqualene desaturase	2.14	2.28E-11
SAOUHSC_02137	sdcS	Sodium-dependent dicarboxylate transporter SdcS	2.15	7.79E-06
SAOUHSC_01017	purH	Bifunctional purine biosynthesis protein PurH	2.17	2.39E-02
SAOUHSC_01318	SAOUHSC_01318	Putative uncharacterized protein	2.18	1.61E-03
SAOUHSC_02729	SAOUHSC_02729	"Amino acid ABC transporter-like protein, putative"	2.20	2.19E-02
SAOUHSC_01603	SAOUHSC_01603	Putative uncharacterized protein	2.21	2.71E-02
SAOUHSC_01921	SAOUHSC_01921	Putative uncharacterized protein	2.21	1.54E-03
SAOUHSC_02822	fbp	"Fructose-1,6-bisphosphatase class 3"	2.31	4.20E-02
SAOUHSC_00160	SAOUHSC_00160	Putative uncharacterized protein	2.31	3.00E-02

SAOUHSC_00291	SAOUHSC_00291	"PfkB family carbohydrate kinase family, putative"	2.32	2.99E-02
SAOUHSC_02772	SAOUHSC_02772	Putative uncharacterized protein	2.32	6.38E-06
SAOUHSC_03028	SAOUHSC_03028	Putative uncharacterized protein	2.34	1.12E-04
SAOUHSC_01920	SAOUHSC_01920	Putative uncharacterized protein	2.35	1.72E-04
SAOUHSC_00061	SAOUHSC_00061	Putative uncharacterized protein	2.35	8.91E-11
SAOUHSC_02425	SAOUHSC_02425	UPF0457 protein SAOUHSC_02425	2.39	1.50E-02
SAOUHSC_00156	SAOUHSC_00156	Putative uncharacterized protein	2.47	5.99E-03
SAOUHSC_02908	SAOUHSC_02908	Putative uncharacterized protein	2.49	3.11E-13
SAOUHSC_02815	SAOUHSC_02815	Putative uncharacterized protein	2.63	1.37E-03
SAOUHSC_01602	SAOUHSC_01602	"Transcriptional regulator, putative"	2.67	2.41E-03
SAOUHSC_01601	SAOUHSC_01601	"Alpha-glucosidase, putative"	2.70	2.45E-03
SAOUHSC_00624	SAOUHSC_00624	"Integrase/recombinase, putative"	2.72	4.42E-10
SAOUHSC_00317	SAOUHSC_00317	Glycerol-3-phosphate transporter	2.81	2.54E-05
SAOUHSC_00196	SAOUHSC_00196	Putative uncharacterized protein	2.82	3.92E-02
SAOUHSC_02882	crtO	"Glycosyl-4,4'-diaponeurosporenoate acyltransferase"	2.93	3.13E-03
SAOUHSC_02771	SAOUHSC_02771	Putative uncharacterized protein	2.96	2.96E-06
SAOUHSC_00846	SAOUHSC_00846	Putative uncharacterized protein	2.97	4.87E-17
SAOUHSC_02812	SAOUHSC_02812	Putative uncharacterized protein	3.00	1.36E-16
SAOUHSC_02881	crtP	Diapolycopene oxygenase	3.08	1.46E-03
SAOUHSC_00157	murQ	N-acetylmuramic acid 6-phosphate etherase	3.13	2.71E-03
SAOUHSC_01910	pckA	Phosphoenolpyruvate carboxykinase [ATP]	3.14	2.47E-03
SAOUHSC_00070	sarS	HTH-type transcriptional regulator SarS	3.26	5.67E-06
SAOUHSC_02403	mtlD	Mannitol-1-phosphate 5-dehydrogenase	3.27	2.51E-06
SAOUHSC_02387	SAOUHSC_02387	Putative uncharacterized protein	3.30	1.70E-22
SAOUHSC_02400	SAOUHSC_02400	"PTS system, mannitol-specific component, putative"	3.31	6.11E-04
SAOUHSC_00158	SAOUHSC_00158	PTS system EIIBC component SAOUHSC_00158	3.39	1.23E-03
SAOUHSC_02879	crtM	Dehydrosqualene synthase	3.46	7.53E-23
SAOUHSC_00831	SAOUHSC_00831	Organic hydroperoxide resistance protein-like	3.48	4.53E-14
SAOUHSC_02401	SAOUHSC_02401	Putative uncharacterized protein	3.63	2.55E-04
SAOUHSC_00069	spa	Immunoglobulin G-binding protein A	3.70	3.06E-06
SAOUHSC_00183	SAOUHSC_00183	"Membrane protein, putative"	3.72	4.68E-05
SAOUHSC_01794	SAOUHSC_01794	"Glyceraldehyde-3-phosphate dehydrogenase, type I"	3.77	2.50E-06
SAOUHSC_02880	crtQ	"4,4'-diaponeurosporenoate glycosyltransferase"	3.88	2.54E-12
SAOUHSC_00356	SAOUHSC_00356	Putative uncharacterized protein	3.96	1.13E-25
SAOUHSC_02402	SAOUHSC_02402	"PTS system, mannitol-specific IIa component, putative"	4.08	7.79E-06
SAOUHSC_00358	SAOUHSC_00358	Putative uncharacterized protein	4.67	3.86E-41
SAOUHSC_02444	SAOUHSC_02444	"Osmoprotectant transporter, BCCT family, opuD-like protein,	4.99	1.19E-39
SAOUHSC_A00354	SAOUHSC_00358.1	Putative uncharacterized protein	5.17	3.74E-17
SAOUHSC_02862	clpL	ATP-dependent Clp protease ATP-binding subunit ClpL	5.24	1.68E-66
SAOUHSC_00619	SAOUHSC_00619	Putative uncharacterized protein	5.66	1.56E-60
SAOUHSC_01730	SAOUHSC_01730	UPF0337 protein SAOUHSC_01730	6.31	4.25E-33
SAOUHSC_01729	SAOUHSC_01729	Putative uncharacterized protein	7.55	3.12E-31
SAOUHSC_02442	SAOUHSC_02442	Putative uncharacterized protein	7.60	4.64E-86
SAOUHSC_02443	SAOUHSC_02443	Putative uncharacterized protein	8.17	7.15E-89
SAOUHSC_00845	SAOUHSC_00845	UPF0337 protein SAOUHSC_00845	8.90	8.31E-61
SAOUHSC_02441	asp23	Alkaline shock protein 23	9.56	2.20E-91

*** Genes highlighted in grey correspond to genes dysregulated in this lysogen but not in the phi80alpha lysogenic strain**

Table S2. Full list of genes dysregulated in biofilms formed by the lysogenic strain RN450-Φ80α compared to the non-lysogenic strain RN

Gene ID*	Gene name	Gene product	Fold-change	padj
SAOUHSC_02260	hld	Delta-hemolysin	-25.60	2.36E-92
SAOUHSC_01135	SAOUHSC_01135	Putative uncharacterized protein	-17.63	4.45E-106
SAOUHSC_01136	SAOUHSC_01136	Putative uncharacterized protein	-16.54	1.11E-100
SAOUHSC_02163			-11.84	5.09E-20
SAOUHSC_02240			-9.04	3.86E-18
SAOUHSC_02907	SAOUHSC_02907	Putative uncharacterized protein	-7.37	7.77E-32
SAOUHSC_02906	SAOUHSC_02906	Putative uncharacterized protein	-7.29	2.90E-26
SAOUHSC_02905	SAOUHSC_02905	Putative uncharacterized protein	-6.71	3.86E-32
SAOUHSC_00401	SAOUHSC_00401	Putative uncharacterized protein	-6.40	2.49E-10
SAOUHSC_02262	SAOUHSC_02262	Putative uncharacterized protein	-6.04	9.89E-47
SAOUHSC_02261	agrB	Accessory gene regulator protein B	-5.98	9.95E-55
SAOUHSC_02264	SAOUHSC_02264	Accessory gene regulator protein C	-5.60	2.13E-53
SAOUHSC_02970			-5.20	2.91E-18
SAOUHSC_02265	SAOUHSC_02265	Accessory gene regulator protein A	-5.03	5.09E-71
SAOUHSC_02969	arcA	Arginine deiminase	-4.59	4.43E-21
SAOUHSC_02967	SAOUHSC_02967	"Arginine/ornithine antiporter, putative"	-4.55	1.80E-36
SAOUHSC_02968	argF	Ornithine carbamoyltransferase	-4.51	1.91E-25
SAOUHSC_02965	arcC2	Carbamate kinase 2	-4.25	3.20E-25
SAOUHSC_02964	arcR	HTH-type transcriptional regulator ArcR	-4.19	3.74E-35
SAOUHSC_01953	SAOUHSC_01953	"Gallidermin superfamily epiA, putative"	-4.17	4.26E-23
SAOUHSC_02266	SAOUHSC_02266	Putative uncharacterized protein	-4.17	1.82E-40
SAOUHSC_02620	SAOUHSC_02620	Putative uncharacterized protein	-4.09	3.78E-31
SAOUHSC_01584	SAOUHSC_01584	Putative uncharacterized protein	-4.03	9.78E-44
SAOUHSC_01942	splA	Serine protease SplA	-3.91	1.68E-07
SAOUHSC_01583	SAOUHSC_01583	Conserved hypothetical phage protein	-3.89	8.70E-36
SAOUHSC_01513	SAOUHSC_01513	Putative uncharacterized protein	-3.80	5.79E-21
SAOUHSC_01512	SAOUHSC_01512	Putative uncharacterized protein	-3.77	3.14E-26
SAOUHSC_00256	SAOUHSC_00256	Putative uncharacterized protein	-3.74	7.77E-32
SAOUHSC_02462	SAOUHSC_02462	Putative uncharacterized protein	-3.69	3.21E-02
SAOUHSC_00257	SAOUHSC_00257	Putative uncharacterized protein	-3.59	1.73E-15
SAOUHSC_01511	SAOUHSC_01511	Putative uncharacterized protein	-3.43	5.25E-19
SAOUHSC_02241	SAOUHSC_02241	Uncharacterized leukocidin-like protein 1	-3.40	1.27E-25
SAOUHSC_02971	SAOUHSC_02971	"Aureolysin, putative"	-3.37	1.69E-32
SAOUHSC_01510	SAOUHSC_01510	Putative uncharacterized protein	-3.37	2.94E-29
SAOUHSC_00272	SAOUHSC_00272	Putative uncharacterized protein	-3.36	2.99E-09
SAOUHSC_01952	SAOUHSC_01952	"Lantibiotic epidermin biosynthesis protein EpiB, putative"	-3.31	2.37E-24
SAOUHSC_01951	SAOUHSC_01951	"Epidermin biosynthesis protein EpiC, authentic point mutatio	-3.24	6.66E-23
SAOUHSC_01508	SAOUHSC_01508	Putative uncharacterized protein	-3.24	1.09E-25
SAOUHSC_02243	SAOUHSC_02243	Uncharacterized leukocidin-like protein 2	-3.20	1.11E-28
SAOUHSC_01950	SAOUHSC_01950	"Flavoprotein, epiD, putative"	-3.15	1.70E-25
SAOUHSC_00988	sspA	Glutamyl endopeptidase	-3.11	8.34E-10
SAOUHSC_01941	splB	Serine protease SplB	-3.10	6.38E-05
SAOUHSC_00274	SAOUHSC_00274	Putative uncharacterized protein	-3.05	5.24E-13
SAOUHSC_00259	SAOUHSC_00259	Putative uncharacterized protein	-3.03	6.68E-12
SAOUHSC_00258	SAOUHSC_00258	Putative uncharacterized protein	-3.03	7.93E-16
SAOUHSC_00260	SAOUHSC_00260	Putative uncharacterized protein	-2.94	1.47E-12
SAOUHSC_00267	SAOUHSC_00267	Putative uncharacterized protein	-2.87	1.64E-19
SAOUHSC_00266	SAOUHSC_00266	Putative uncharacterized protein	-2.85	2.64E-22
SAOUHSC_02709	hlgC	Gamma-hemolysin component C	-2.85	1.58E-09
SAOUHSC_01949	SAOUHSC_01949	"Intracellular serine protease, putative"	-2.78	2.01E-15
SAOUHSC_00262	SAOUHSC_00262	Putative uncharacterized protein	-2.75	3.54E-09

SAOUHSC_00261	SAOUHSC_00261	Putative uncharacterized protein	-2.75	5.72E-11
SAOUHSC_02161	SAOUHSC_02161	MHC class II analog protein	-2.72	5.19E-03
SAOUHSC_00264	SAOUHSC_00264	Putative uncharacterized protein	-2.72	1.81E-08
SAOUHSC_02706	sbi	Immunoglobulin-binding protein sbi	-2.69	1.12E-03
SAOUHSC_01944	SAOUHSC_01944	Putative uncharacterized protein	-2.65	2.97E-04
SAOUHSC_00961	SAOUHSC_00961	Putative uncharacterized protein	-2.63	8.10E-08
SAOUHSC_00265	SAOUHSC_00265	Putative uncharacterized protein	-2.60	1.72E-09
SAOUHSC_00717	SAOUHSC_00717	Putative uncharacterized protein	-2.59	8.28E-03
SAOUHSC_00275	SAOUHSC_00275	Putative uncharacterized protein	-2.57	5.72E-09
SAOUHSC_00280	SAOUHSC_00280	Putative uncharacterized protein	-2.55	2.68E-14
SAOUHSC_T0003			-2.53	3.24E-03
SAOUHSC_00055	SAOUHSC_00055	Uncharacterized lipoprotein SAOUHSC_00055	-2.50	1.88E-16
SAOUHSC_A01912	SAOUHSC_01993.3	Putative uncharacterized protein	-2.46	8.49E-03
SAOUHSC_01945	SAOUHSC_01945	"Membrane protein, putative"	-2.46	1.27E-13
SAOUHSC_00561	SAOUHSC_00561	Putative uncharacterized protein	-2.46	1.88E-16
SAOUHSC_00054	SAOUHSC_00054	Uncharacterized lipoprotein SAOUHSC_00054	-2.45	3.30E-12
SAOUHSC_00053	SAOUHSC_00053	Uncharacterized lipoprotein SAOUHSC_00053	-2.43	1.11E-16
SAOUHSC_02707	SAOUHSC_02707	Putative uncharacterized protein	-2.40	3.17E-02
SAOUHSC_01121	hly	Alpha-hemolysin	-2.38	3.50E-05
SAOUHSC_02795	SAOUHSC_02795	Putative uncharacterized protein	-2.38	5.58E-10
SAOUHSC_02710	hlgB	Gamma-hemolysin component B	-2.37	4.00E-13
SAOUHSC_01948	SAOUHSC_01948	ABC transporter domain protein	-2.36	6.55E-17
SAOUHSC_02872	SAOUHSC_02872	Putative uncharacterized protein	-2.36	3.93E-04
SAOUHSC_02108	ftnA	Ferritin	-2.34	1.30E-02
SAOUHSC_01947	SAOUHSC_01947	"Membrane protein, putative"	-2.33	3.30E-18
SAOUHSC_00986	sspC	Staphostatin B	-2.29	8.68E-05
SAOUHSC_T00029			-2.29	5.69E-03
SAOUHSC_00279	SAOUHSC_00279	Putative uncharacterized protein	-2.27	2.33E-12
SAOUHSC_00987	sspB	Staphopain B	-2.26	5.87E-04
SAOUHSC_02312	kdpA	Potassium-transporting ATPase A chain	-2.25	1.06E-04
SAOUHSC_02452	lacD	"Tagatose 1,6-diphosphate aldolase"	-2.23	4.99E-04
SAOUHSC_00052	SAOUHSC_00052	Uncharacterized lipoprotein SAOUHSC_00052	-2.23	2.36E-12
SAOUHSC_02109	SAOUHSC_02109	Putative uncharacterized protein	-2.22	2.34E-02
SAOUHSC_01936	splE	Serine protease SplE	-2.21	2.89E-05
SAOUHSC_02692	SAOUHSC_02692	Putative uncharacterized protein	-2.21	6.50E-04
SAOUHSC_00807	SAOUHSC_00807	Putative uncharacterized protein	-2.21	2.93E-12
SAOUHSC_00975	SAOUHSC_00975	Putative uncharacterized protein	-2.20	3.16E-07
SAOUHSC_00047	SAOUHSC_00047	Putative uncharacterized protein	-2.19	2.06E-10
SAOUHSC_00818	SAOUHSC_00818	Thermonuclease	-2.17	4.60E-07
SAOUHSC_00277	SAOUHSC_00277	Putative uncharacterized protein	-2.16	4.87E-11
SAOUHSC_00918	SAOUHSC_00918	Truncated MHC class II analog protein	-2.14	3.54E-10
SAOUHSC_00276	SAOUHSC_00276	Putative uncharacterized protein	-2.14	1.16E-06
SAOUHSC_03003	icaD	"Poly-beta-1,6-N-acetyl-D-glucosamine synthesis protein IcaD'	-2.14	1.69E-03
SAOUHSC_02794	SAOUHSC_02794	Putative uncharacterized protein	-2.13	3.17E-07
SAOUHSC_00808	SAOUHSC_00808	Putative uncharacterized protein	-2.13	2.62E-12
SAOUHSC_01939	splC	Serine protease SplC	-2.12	3.12E-05
SAOUHSC_01935	splF	Serine protease SplF	-2.11	9.33E-05
SAOUHSC_02160	SAOUHSC_02160	Putative uncharacterized protein	-2.10	3.73E-02
SAOUHSC_02865	SAOUHSC_02865	Putative uncharacterized protein	-2.09	2.53E-09
SAOUHSC_01180	SAOUHSC_01180	Putative uncharacterized protein	-2.09	9.16E-12
SAOUHSC_02691	SAOUHSC_02691	Putative uncharacterized protein	-2.07	3.38E-03
SAOUHSC_00268	SAOUHSC_00268	Putative uncharacterized protein	-2.06	7.68E-12

SAOUHSC_02451	SAOUHSC_02451	"PTS system lactose-specific IIA component, putative"	-2.06	1.05E-02
SAOUHSC_03004	icaB	"Poly-beta-1,6-N-acetyl-D-glucosamine N-deacetylase"	-2.05	7.71E-05
SAOUHSC_02999	SAOUHSC_02999	Capsular polysaccharide biosynthesis protein Cap5B	-2.04	1.66E-05
SAOUHSC_00806	SAOUHSC_00806	Putative uncharacterized protein	-2.04	2.93E-11
SAOUHSC_02453	lacC	Tagatose-6-phosphate kinase	-2.03	4.34E-05
SAOUHSC_00191	SAOUHSC_00191	Putative uncharacterized protein	-2.02	2.09E-02
SAOUHSC_03000	SAOUHSC_03000	"Capsular polysaccharide biosynthesis, capA, putative"	-2.00	2.55E-03
SAOUHSC_00716	SAOUHSC_00716	Putative uncharacterized protein	-2.00	2.78E-02
SAOUHSC_03023	drp35	Lactonase drp35	2.01	9.53E-05
SAOUHSC_01014	SAOUHSC_01014	Amidophosphoribosyltransferase	2.01	3.90E-02
SAOUHSC_02763	SAOUHSC_02763	"Peptide ABC transporter, ATP-binding protein, putative"	2.01	6.83E-07
SAOUHSC_02820	SAOUHSC_02820	Putative uncharacterized protein	2.01	1.26E-08
SAOUHSC_02767	SAOUHSC_02767	"Peptide ABC transporter, peptide-binding protein, putative"	2.01	5.23E-05
SAOUHSC_01087	SAOUHSC_01087	"Iron compound ABC transporter, permease protein"	2.02	1.16E-03
SAOUHSC_02994	SAOUHSC_02994	Putative uncharacterized protein	2.02	1.06E-11
SAOUHSC_01088	SAOUHSC_01088	Putative uncharacterized protein	2.02	7.59E-04
SAOUHSC_00532	SAOUHSC_00532	Putative uncharacterized protein	2.03	4.41E-03
SAOUHSC_01082	isdC	Iron-regulated surface determinant protein C	2.03	1.17E-03
SAOUHSC_01079	isdB	Iron-regulated surface determinant protein B	2.03	2.93E-09
SAOUHSC_00155	ptsG	PTS system glucose-specific EIICBA component	2.04	9.04E-13
SAOUHSC_02830	SAOUHSC_02830	"D-lactate dehydrogenase, putative"	2.05	3.89E-08
SAOUHSC_02330	SAOUHSC_02330	Phosphomethylpyrimidine kinase	2.05	1.83E-13
SAOUHSC_00205	SAOUHSC_00205	Putative uncharacterized protein	2.06	3.77E-03
SAOUHSC_00604	SAOUHSC_00604	Putative uncharacterized protein	2.06	1.44E-03
SAOUHSC_00139	SAOUHSC_00139	Putative uncharacterized protein	2.07	2.46E-03
SAOUHSC_02466	SAOUHSC_02466	Truncated MHC class II analog protein	2.07	3.50E-07
SAOUHSC_00748	SAOUHSC_00748	Putative uncharacterized protein	2.08	1.67E-06
SAOUHSC_02331	tenA	Putative thiaminase-2	2.08	2.25E-14
SAOUHSC_00153	SAOUHSC_00153	"Indolepyruvate decarboxylase, putative"	2.09	1.35E-02
SAOUHSC_02936	SAOUHSC_02936	Putative uncharacterized protein	2.11	5.93E-06
SAOUHSC_00152	SAOUHSC_00152	Putative uncharacterized protein	2.12	1.10E-02
SAOUHSC_01854	SAOUHSC_01854	Putative uncharacterized protein	2.12	9.48E-15
SAOUHSC_02821	SAOUHSC_02821	"Membrane spanning protein, putative"	2.13	1.77E-10
SAOUHSC_00737	SAOUHSC_00737	Putative uncharacterized protein	2.13	3.54E-03
SAOUHSC_00848	SAOUHSC_00848	Putative uncharacterized protein	2.15	2.19E-09
SAOUHSC_02363	SAOUHSC_02363	Putative aldehyde dehydrogenase SAOUHSC_02363	2.17	8.45E-08
SAOUHSC_00371	SAOUHSC_00371	Putative uncharacterized protein	2.17	3.44E-04
SAOUHSC_02754	SAOUHSC_02754	"ABC transporter, ATP-binding protein, putative"	2.18	2.13E-03
SAOUHSC_00291	SAOUHSC_00291	"PfkB family carbohydrate kinase family, putative"	2.18	5.68E-03
SAOUHSC_00749	SAOUHSC_00749	Putative uncharacterized protein	2.19	3.21E-10
SAOUHSC_01855	SAOUHSC_01855	UPF0478 protein SAOUHSC_01855	2.22	1.02E-19
SAOUHSC_02975	SAOUHSC_02975	"PTS system, fructose-specific IIABC component, putative"	2.22	1.86E-03
SAOUHSC_02329	thiM	Hydroxyethylthiazole kinase	2.23	1.85E-13
SAOUHSC_00895	SAOUHSC_00895	Glutamate dehydrogenase	2.23	3.60E-04
SAOUHSC_01847	SAOUHSC_01847	Putative uncharacterized protein	2.24	4.98E-03
SAOUHSC_02663	SAOUHSC_02663	Putative uncharacterized protein	2.24	4.44E-04
SAOUHSC_02803	fnbA	Fibronectin-binding protein A	2.25	5.29E-09
SAOUHSC_00285	SAOUHSC_00285	Putative uncharacterized protein	2.26	3.68E-12
SAOUHSC_00690	SAOUHSC_00690	Putative uncharacterized protein	2.26	7.59E-18
SAOUHSC_01849	SAOUHSC_01849	Putative uncharacterized protein	2.26	9.57E-04
SAOUHSC_03006	lipA	Lipase 1	2.29	2.09E-03
SAOUHSC_01416	odhB	Dihydrolipoyllysine-residue succinyltransferase component of	2.29	3.42E-04

SAOUHSC_00180	SAOUHSC_00180	Putative uncharacterized protein	2.30	1.01E-02
SAOUHSC_02608	SAOUHSC_02608	Putative uncharacterized protein	2.31	1.24E-03
SAOUHSC_00847	SAOUHSC_00847	"ABC transporter, ATP-binding protein, putative"	2.31	1.95E-16
SAOUHSC_01844	SAOUHSC_01844	Putative uncharacterized protein	2.31	4.94E-02
SAOUHSC_02661	SAOUHSC_02661	"PTS system sucrose-specific IIBC component, putative"	2.33	7.38E-14
SAOUHSC_02409	SAOUHSC_02409	Arginase	2.34	2.42E-02
SAOUHSC_01015	purM	Phosphoribosylformylglycinamidine cyclo-ligase	2.34	3.00E-02
SAOUHSC_01027	SAOUHSC_01027	Putative uncharacterized protein	2.35	4.50E-14
SAOUHSC_01922	SAOUHSC_01922	Putative uncharacterized protein	2.36	9.54E-09
SAOUHSC_01018	purD	Phosphoribosylamine--glycine ligase	2.43	2.75E-06
SAOUHSC_02013	SAOUHSC_02013	Uncharacterized protein SAOUHSC_02013	2.43	1.98E-19
SAOUHSC_00747	SAOUHSC_00747	Putative uncharacterized protein	2.45	4.87E-12
SAOUHSC_02662	SAOUHSC_02662	PTS system sucrose-specific IIBC component	2.46	2.00E-19
SAOUHSC_01275	SAOUHSC_01275	Putative uncharacterized protein	2.48	1.73E-03
SAOUHSC_01318	SAOUHSC_01318	Putative uncharacterized protein	2.48	4.04E-05
SAOUHSC_01016	SAOUHSC_01016	"Phosphoribosylglycinamide formyltransferase, putative"	2.51	1.99E-02
SAOUHSC_00296	SAOUHSC_00296	ROK family protein	2.51	3.38E-02
SAOUHSC_00658	SAOUHSC_00658	Putative uncharacterized protein	2.53	1.40E-03
SAOUHSC_00294	SAOUHSC_00294	Putative uncharacterized protein	2.54	3.90E-02
SAOUHSC_01017	purH	Bifunctional purine biosynthesis protein PurH	2.57	7.82E-04
SAOUHSC_01418	odhA	2-oxoglutarate dehydrogenase E1 component	2.57	3.40E-05
SAOUHSC_02774	SAOUHSC_02774	Putative uncharacterized protein	2.60	1.24E-11
SAOUHSC_00746	SAOUHSC_00746	Putative uncharacterized protein	2.62	2.50E-08
SAOUHSC_00656	SAOUHSC_00656	Putative uncharacterized protein	2.64	4.91E-03
SAOUHSC_00310	SAOUHSC_00310	Putative uncharacterized protein	2.66	2.15E-03
SAOUHSC_00894	rocD	Ornithine aminotransferase	2.66	1.66E-04
SAOUHSC_02339	SAOUHSC_02339	Putative uncharacterized protein	2.67	3.51E-05
SAOUHSC_02899	SAOUHSC_02899	Putative uncharacterized protein	2.68	1.37E-23
SAOUHSC_02607	hutU	Urocanate hydratase	2.72	2.66E-03
SAOUHSC_00037	SAOUHSC_00037	Putative uncharacterized protein	2.72	1.19E-21
SAOUHSC_01921	SAOUHSC_01921	Putative uncharacterized protein	2.74	4.33E-09
SAOUHSC_00655	SAOUHSC_00655	Putative uncharacterized protein	2.76	9.29E-04
SAOUHSC_00199	SAOUHSC_00199	Acetate CoA-transferase YdiF	2.76	1.60E-03
SAOUHSC_01918	SAOUHSC_01918	Putative uncharacterized protein	2.89	1.22E-06
SAOUHSC_01603	SAOUHSC_01603	Putative uncharacterized protein	2.89	3.31E-04
SAOUHSC_02848	glcB	PTS system glucoside-specific EIICBA component	2.90	2.77E-13
SAOUHSC_02597	SAOUHSC_02597	"PTS system component, putative"	2.90	9.53E-06
SAOUHSC_00383	SAOUHSC_00383	Putative uncharacterized protein	2.90	1.03E-11
SAOUHSC_00309	SAOUHSC_00309	Putative uncharacterized protein	2.92	3.45E-32
SAOUHSC_00071	SAOUHSC_00071	"Lipoprotein, SirC, putative"	2.97	1.76E-21
SAOUHSC_03035	SAOUHSC_03035	Putative uncharacterized protein	3.01	1.98E-23
SAOUHSC_02610	hutG	Formimidoylglutamase	3.02	1.95E-23
SAOUHSC_02925	SAOUHSC_02925	Putative uncharacterized protein	3.06	2.90E-08
SAOUHSC_02097	SAOUHSC_02097	Putative uncharacterized protein	3.12	3.23E-21
SAOUHSC_02729	SAOUHSC_02729	"Amino acid ABC transporter-like protein, putative"	3.14	6.25E-05
SAOUHSC_02665	SAOUHSC_02665	Putative uncharacterized protein	3.14	1.54E-18
SAOUHSC_01919	SAOUHSC_01919	Putative uncharacterized protein	3.15	3.38E-08
SAOUHSC_02137	sdcS	Sodium-dependent dicarboxylate transporter SdcS	3.17	4.72E-17
SAOUHSC_02822	fbp	"Fructose-1,6-bisphosphatase class 3"	3.17	1.14E-03
SAOUHSC_00156	SAOUHSC_00156	Putative uncharacterized protein	3.18	4.41E-05
SAOUHSC_01920	SAOUHSC_01920	Putative uncharacterized protein	3.23	1.38E-15
SAOUHSC_02425	SAOUHSC_02425	UPF0457 protein SAOUHSC_02425	3.26	4.86E-05

SAOUHSC_02799	sarT	HTH-type transcriptional regulator SarT	3.35	5.41E-05
SAOUHSC_02753	SAOUHSC_02753	"Membrane protein, putative"	3.38	1.44E-13
SAOUHSC_00632	mnhG2	Putative antiporter subunit mnhG2	3.42	8.90E-17
SAOUHSC_00160	SAOUHSC_00160	Putative uncharacterized protein	3.46	2.96E-07
SAOUHSC_02930	SAOUHSC_02930	Putative uncharacterized protein	3.46	4.53E-16
SAOUHSC_00630	SAOUHSC_00630	Putative uncharacterized protein	3.51	1.99E-20
SAOUHSC_00629	mnhE2	Putative antiporter subunit mnhE2	3.58	2.47E-15
SAOUHSC_02869	rocA	1-pyrroline-5-carboxylate dehydrogenase	3.59	4.63E-04
SAOUHSC_00198	SAOUHSC_00198	Putative uncharacterized protein	3.66	6.96E-04
SAOUHSC_A02680	SAOUHSC_02822.1	Putative uncharacterized protein	3.69	7.84E-04
SAOUHSC_00626	mnhB2	Putative antiporter subunit mnhB2	3.70	2.15E-15
SAOUHSC_00628	mnhD2	Putative antiporter subunit mnhD2	3.75	2.23E-25
SAOUHSC_00317	SAOUHSC_00317	Glycerol-3-phosphate transporter	3.78	6.90E-10
SAOUHSC_00736	SAOUHSC_00736	Putative lipid kinase SAOUHSC_00736	3.78	3.09E-28
SAOUHSC_00812	clfA	Clumping factor A	3.82	4.54E-23
SAOUHSC_00627	mnhC2	Putative antiporter subunit mnhC2	3.97	1.02E-19
SAOUHSC_02863	SAOUHSC_02863	Putative uncharacterized protein	3.97	1.68E-30
SAOUHSC_01602	SAOUHSC_01602	"Transcriptional regulator, putative"	3.98	3.59E-06
SAOUHSC_01024	SAOUHSC_01024	Putative uncharacterized protein	4.03	5.53E-06
SAOUHSC_01601	SAOUHSC_01601	"Alpha-glucosidase, putative"	4.10	1.90E-07
SAOUHSC_00196	SAOUHSC_00196	Putative uncharacterized protein	4.12	4.20E-04
SAOUHSC_00157	murQ	N-acetylmuramic acid 6-phosphate etherase	4.18	1.77E-06
SAOUHSC_01846	SAOUHSC_01846	"Acetyl-CoA synthetase, putative"	4.19	4.25E-05
SAOUHSC_00625	mnhA2	Putative antiporter subunit mnhA2	4.29	4.23E-46
SAOUHSC_00569	SAOUHSC_00569	Putative uncharacterized protein	4.33	7.03E-40
SAOUHSC_00158	SAOUHSC_00158	PTS system EIIBC component SAOUHSC_00158	4.35	1.09E-07
SAOUHSC_01884	SAOUHSC_01884	Putative uncharacterized protein	4.36	1.36E-04
SAOUHSC_00179	SAOUHSC_00179	Putative uncharacterized protein	4.37	1.80E-04
SAOUHSC_00825	SAOUHSC_00825	Putative uncharacterized protein	4.37	2.78E-36
SAOUHSC_02815	SAOUHSC_02815	Putative uncharacterized protein	4.38	2.90E-08
SAOUHSC_02900	SAOUHSC_02900	Uncharacterized hydrolase SAOUHSC_02900	4.43	2.82E-60
SAOUHSC_03032	SAOUHSC_03032	Putative uncharacterized protein	4.46	6.47E-21
SAOUHSC_02604	SAOUHSC_02604	Putative uncharacterized protein	4.64	1.19E-64
SAOUHSC_00070	sarS	HTH-type transcriptional regulator SarS	4.73	7.12E-10
SAOUHSC_02809	SAOUHSC_02809	"Gluconate operon transcriptional repressor, putative"	4.82	2.57E-06
SAOUHSC_02440	SAOUHSC_02440	Putative uncharacterized protein	4.88	1.12E-04
SAOUHSC_01910	pckA	Phosphoenolpyruvate carboxykinase [ATP]	4.92	7.37E-07
SAOUHSC_00061	SAOUHSC_00061	Putative uncharacterized protein	4.97	3.31E-57
SAOUHSC_00175	SAOUHSC_00175	"Multiple sugar-binding transport ATP-binding protein, putativ	5.19	4.97E-06
SAOUHSC_00311	SAOUHSC_00311	Putative uncharacterized protein	5.24	1.73E-04
SAOUHSC_00069	spa	Immunoglobulin G-binding protein A	5.40	8.93E-13
SAOUHSC_02771	SAOUHSC_02771	Putative uncharacterized protein	5.46	2.23E-18
SAOUHSC_01794	SAOUHSC_01794	"Glyceraldehyde-3-phosphate dehydrogenase, type I"	5.80	6.92E-11
SAOUHSC_02908	SAOUHSC_02908	Putative uncharacterized protein	5.89	8.09E-49
SAOUHSC_00826	SAOUHSC_00826	Putative uncharacterized protein	5.92	5.39E-51
SAOUHSC_02877	crtN	Dehydrosqualene desaturase	5.93	2.91E-51
SAOUHSC_02808	SAOUHSC_02808	Gluconate kinase	6.39	6.98E-03
SAOUHSC_03028	SAOUHSC_03028	Putative uncharacterized protein	6.42	3.48E-34
SAOUHSC_02772	SAOUHSC_02772	Putative uncharacterized protein	6.49	7.77E-32
SAOUHSC_00312	SAOUHSC_00312	Putative uncharacterized protein	6.73	2.78E-02
SAOUHSC_02806	SAOUHSC_02806	"Gluconate permease, putative"	7.11	2.21E-08
SAOUHSC_00624	SAOUHSC_00624	"Integrase/recombinase, putative"	7.23	2.68E-57

SAOUHSC_00183	SAOUHSC_00183	"Membrane protein, putative"	7.31	1.18E-15
SAOUHSC_00176	SAOUHSC_00176	"Bacterial extracellular solute-binding protein, putative"	7.77	2.08E-02
SAOUHSC_02812	SAOUHSC_02812	Putative uncharacterized protein	7.98	1.32E-72
SAOUHSC_02400	SAOUHSC_02400	"PTS system, mannitol-specific component, putative"	8.23	1.50E-13
SAOUHSC_00177	SAOUHSC_00177	"Maltose ABC transporter, permease protein, putative"	8.41	7.15E-03
SAOUHSC_00846	SAOUHSC_00846	Putative uncharacterized protein	8.59	6.06E-82
SAOUHSC_00178	SAOUHSC_00178	"Maltose ABC transporter, permease protein"	8.60	3.24E-03
SAOUHSC_02403	mtlD	Mannitol-1-phosphate 5-dehydrogenase	8.74	2.87E-23
SAOUHSC_02882	crtO	"Glycosyl-4,4'-diaponeurosporenoate acyltransferase"	8.93	4.56E-14
SAOUHSC_00831	SAOUHSC_00831	Organic hydroperoxide resistance protein-like	9.45	2.42E-60
SAOUHSC_02401	SAOUHSC_02401	Putative uncharacterized protein	9.77	1.99E-14
SAOUHSC_02881	crtP	Diapolycopene oxygenase	10.10	1.13E-13
SAOUHSC_02879	crtM	Dehydrosqualene synthase	10.17	6.60E-88
SAOUHSC_02402	SAOUHSC_02402	"PTS system, mannitol-specific IIa component, putative"	11.06	3.94E-20
SAOUHSC_02387	SAOUHSC_02387	Putative uncharacterized protein	11.15	5.60E-97
SAOUHSC_00356	SAOUHSC_00356	Putative uncharacterized protein	11.49	2.50E-66
SAOUHSC_00358	SAOUHSC_00358	Putative uncharacterized protein	12.22	7.92E-85
SAOUHSC_02880	crtQ	"4,4'-diaponeurosporenoate glycosyltransferase"	13.11	1.33E-52
SAOUHSC_A00354	SAOUHSC_00358.1	Putative uncharacterized protein	13.93	9.78E-44
SAOUHSC_02862	clpL	ATP-dependent Clp protease ATP-binding subunit ClpL	16.79	1.28E-231
SAOUHSC_01729	SAOUHSC_01729	Putative uncharacterized protein	18.24	1.86E-105
SAOUHSC_01730	SAOUHSC_01730	UPF0337 protein SAOUHSC_01730	19.71	3.15E-197
SAOUHSC_02444	SAOUHSC_02444	"Osmoprotectant transporter, BCCT family, opuD-like protein,	19.92	1.15E-144
SAOUHSC_00619	SAOUHSC_00619	Putative uncharacterized protein	21.10	2.64E-196
SAOUHSC_02442	SAOUHSC_02442	Putative uncharacterized protein	27.27	1.58E-186
SAOUHSC_02441	asp23	Alkaline shock protein 23	28.18	2.18E-286
SAOUHSC_02443	SAOUHSC_02443	Putative uncharacterized protein	29.88	1.28E-231
SAOUHSC_00845	SAOUHSC_00845	UPF0337 protein SAOUHSC_00845	31.38	5.63E-205

*** Genes highlighted in grey correspond to genes dysregulated in this lysogen but not in the phi11 lysogenic strain**

Table S3. Normalized mean reads per kilobase million (RPKM) values corresponding to the different open reading frames (ORFs) of Φ11 genome in samples of strain RN450-Φ11

gene_ID	start_coord	end_coord	phi11-1 Reads	phi11-2 Reads	phi11-3 Reads	phi11-1 RPKM	phi11-2 RPKM	phi11-3 RPKM	RPKM MEAN	RPKM ESTAND.	gene_name
phi11_01	83	1129	2506	2033	1083	12992	9385	6630	**9669**	3190.494162	integrase
phi11_02	1241	1441	896	822	569	24196	19765	18144	**20701.66667**	3132.83966	excisionase
phi11_03	1378	2283	10055	9648	6842	60240	51468	48402	**53370**	6143.920898	PV83 orf 3-like protei
phi11_04	2319	2504	1764	1411	1186	51477	36664	40868	**43003**	7633.800561	PV83 orf 4-like protei
phi11_05	2901	3620	7063	6399	5458	53246	42954	48586	**48262**	5153.644148	cI-like repressor
phi11_06	3762	3980	1307	1493	1062	32394	32949	31081	**32141.33333**	959.289494	cro-like repressor
phi11_07	4427	5251	5420	5923	4727	35660	34699	36723	**35694**	1012.428269	anti-repressor
phi11_08	5252	5476	1262	1222	1082	30444	26249	30822	**29171.66667**	2538.150179	phi11_08
phi11_09	5518	5967	3391	4156	3029	40902	44637	43142	**42893.66667**	1879.842635	phi PV83 orf 10-like p
phi11_10	5981	6202	3158	4051	2895	77213	88194	83581	**82996**	5513.824353	phi PV83 orf 12-like p
phi11_11	6696	7016	473	657	482	7998	9892	9624	**9171.333333**	1024.933819	phi PVL orf 39-like pr
phi11_12	7566	8345	4210	4701	3756	29297	29129	30863	**29763**	956.3242128	phi ETA orf 17-like pr
phi11_13	8375	8929	3324	4191	2975	32509	36497	34356	**34454**	1995.805351	ssb
phi11_14	8942	9634	3489	4338	3094	27327	30254	28615	**28732**	1467.003408	phi PV83 orf 19-like p
phi11_15	9606	10412	3879	4462	3441	26090	26723	27329	**26714**	619.5490295	phi PV83 orf 20-like p
phi11_16	10765	12006	7167	8479	6286	31322	32995	32439	**32252**	852.0322764	helicase DnaB
phi11_17	12222	12443	1146	1316	999	28020	28650	28842	**28504**	430.0093022	phi ETA orf 24-like pr
phi11_18	12454	12858	1524	2095	1519	20425	25001	24039	**23155**	2412.682325	phi ETA orf 25-like pr
phi11_19	12863	13048	844	1122	917	24630	29155	31599	**28461.33333**	3535.904458	PV83 orf 23-like prote
phi11_20	13049	13318	1079	1421	1147	21691	25437	27228	**24785.33333**	2825.437016	HTH DNA binding pro
phi11_21	13319	13678	1168	1494	1276	17610	20058	22717	**20128.33333**	2554.226367	phi PVL orf 50-like pr
phi11_22	13679	13927	1276	1523	1120	27815	29562	28829	**28735.33333**	877.2584188	phi PVL orf 51-like pr
phi11_23	13933	14187	1365	1562	1325	29055	29605	33303	**30654.33333**	2310.238372	phi PVL orf 52-like pr
phi11_24	14184	14426	1082	1300	1120	24169	25856	29541	**26522**	2747.22824	ETA orf 33-like protei
phi11_25	14419	14928	2671	3217	2772	28427	30487	34836	**31250**	3271.917939	dUTPase
phi11_26	15037	15210	407	471	420	12696	13083	15471	**13750**	1502.938122	phi SLT orf 81b-like p
phi11_27	15558	15746	703	872	706	20189	22299	23942	**22143.33333**	1881.336316	rinB
phi11_28	15917	16339	1912	2743	2030	24535	31341	30759	**28878.33333**	3772.67668	rinA
phi11_29	16526	16966	385	686	552	4739	7518	8023	**6760**	1768.357147	small terminase
phi11_30	16887	18230	1581	2634	2267	6385	9472	10811	**8889.333333**	2269.800505	large terminase
phi11_31	18241	19776	2389	3576	2971	8442	11252	12397	**10697**	2035.073709	phi Mu50B-like prote
phi11_32	19894	20778	1302	1930	1840	7985	10540	13326	**10617**	2671.332439	phi Mu50B-like prote
phi11_33	21115	21750	3419	5277	5138	29179	40101	51778	**40352.66667**	11301.60176	phi Mu50B-like prote
phi11_34	21764	22738	4957	8108	6858	27596	40192	45082	**37623.33333**	9021.562245	head protein
phi11_35	22760	23047	874	1562	1304	16472	26213	29020	**23901.66667**	6585.573045	phi Mu50B-like prote
phi11_36	23056	23388	1095	2097	1726	17848	30436	33221	**27168.33333**	8190.880071	phi Mu50B-like prote
phi11_37	23687	24034	1713	2852	2472	26718	39609	45528	**37285**	9617.939332	phi Mu50B-like prote
phi11_38	24046	24429	1522	2280	1859	21514	28697	31028	**27079.66667**	4958.918666	phi Mu50B-like prote
phi11_39	24448	25029	2594	3798	3421	24192	31540	37674	**31135.33333**	6750.103505	structural phi Mu50B
phi11_40	25091	25456	991	1571	1443	14697	20746	25269	**20237.33333**	5304.323928	phi11_40
phi11_41	25486	25830	994	1641	1482	15639	22989	27532	**22053.33333**	6001.455351	phi11_41
phi11_42	26582	29314	8024	11459	9620	15936	20265	22560	**19587**	3363.644898	tape measure protein
phi11_43	29327	30274	2796	3880	3386	16009	19781	22892	**19560.66667**	3446.785797	phi ETA orf 54-like pr
phi11_44	30283	32184	3977	5271	4712	11349	13394	15878	**13540.33333**	2268.043283	phi ETA orf 55-like pr
phi11_45	32199	34109	3926	5698	4762	11151	14411	15971	**13844.33333**	2459.457935	phi ETA orf 56-like pr
phi11_46	35932	36309	532	689	608	7639	8810	10309	**8919.333333**	1338.353591	phi105 orf 44-like pro

phi11_47	36313	36486	211	241	211	6582	6694	7772	**7016**	657.1057754	phi ETA orf 58-like pr
phi11_48	36526	36825	342	550	448	6188	8861	9571	**8206.666667**	1783.896391	phi SLT orf 99-like pr
phi11_49	36962	38860	3200	5130	4143	9147	13056	13983	**12062**	2566.66145	cell wall hydrolase
phi11_50	38873	40045	2234	3125	2565	10337	12876	14015	**12409.33333**	1882.884578	tail fiber
phi11_51	40051	40446	1378	1990	1505	18888	24288	24359	**22511.66667**	3138.388174	phi ETA orf 63-like pr
phi11_52	40502	40939	863	1228	1050	10695	13550	15365	**13203.33333**	2354.221386	holin
phi11_53	40920	42365	3572	4509	3873	13408	15071	17167	**15215.33333**	1883.651861	amidase

Table S4. Normalized mean reads per kilobase million (RPKM) values corresponding to the different open reading frames (ORFs) of $\Phi80\alpha$ genome in samples of strain RN450-$\Phi80\alpha$

gene_ID	start_coord	end_coord	phi80-1 Reads	phi80-2 Reads	phi80-3 Reads	phi80-1 RPKM	phi80-2 RPKM	phi80-3 RPKM	RPKM MEAN	RPKM ESTAND.	gene_name
SPV-80A_gp01	32	1417	17180	8997	6710	59202	34948	26071	**40073.66667**	17149.93103	int
SPV-80A_gp02	1525	1725	5870	4017	4490	139482	107594	120296	**122457.3333**	16053.49362	xis
SPV-80A_gp03	1662	2567	66096	55552	55700	348435	330107	331075	**336539**	10313.60112	hypothetical protein
SPV-80A_gp04	2603	2788	15034	12040	10498	386044	348495	303944	**346161**	41099.73439	hypothetical protein
SPV-80A_gp05	2948	3115	19816	17151	15551	563355	549621	498481	**537152.3333**	34187.12777	hypothetical protein
SPV-80A_gp06	3185	3901	85840	78565	76483	571802	589920	574441	**578721**	9787.966132	CI-like repressor
SPV-80A_gp07	4065	4307	2183	1450	1127	42906	32125	24976	**33335.66667**	9026.101613	cro-like repressor
SPV-80A_gp08	4323	5111	10593	6984	5787	64123	47655	39498	**50425.33333**	12544.07096	antirepressor
SPV-80A_gp09	5127	5321	2347	1680	1349	57485	46383	37254	**47040.66667**	10131.52182	hypothetical protein
SPV-80A_gp10	5374	5550	1400	1208	1193	37777	36743	36297	**36939**	759.2180188	hypothetical protein
SPV-80A_gp11	5525	5755	2510	2584	3177	51896	60223	74064	**62061**	11197.71133	hypothetical protein
SPV-80A_gp12	5814	5942	1286	993	976	47613	41442	40744	**43266.33333**	3780.467476	hypothetical protein
SPV-80A_gp13	5935	6096	983	655	464	28981	21768	15424	**22057.66667**	6783.1403	hypothetical protein
SPV-80A_gp14	6188	6448	1797	1154	1002	32884	23804	20674	**25787.33333**	6342.021234	hypothetical protein
SPV-80A_gp15	6458	6679	1879	1344	1110	40425	32593	26926	**33314.66667**	6778.373871	hypothetical protein
SPV-80A_gp16	6672	7295	4461	3097	2342	34145	26720	20212	**27025.66667**	6971.527547	e12
SPV-80A_gp17	7295	7723	3165	2049	1638	35236	25714	20562	**27170.66667**	7444.660995	ssb
SPV-80A_gp18	7737	8411	5083	3431	2738	35966	27365	21844	**28391.66667**	7116.758696	hypothetical protein
SPV-80A_gp19	8517	9374	33807	37725	44525	188189	236714	279458	**234787**	45665.00396	hypothetical protein
SPV-80A_gp20	9439	10209	3785	3010	2626	23447	21018	18342	**20935.66667**	2553.495709	hypothetical protein
SPV-80A_gp21	10219	10998	3490	2534	2149	21370	17490	14837	**17899**	3285.648033	DnaC
SPV-80A_gp22	10992	11150	703	496	415	21117	16795	14056	**17322.66667**	3559.951451	hypothetical protein
SPV-80A_gp23	11163	11384	996	696	579	21428	16879	14045	**17450.66667**	3724.550219	hypothetical protein
SPV-80A_gp24	11394	11801	1337	1016	890	15651	13407	11747	**13601.66667**	1959.266529	RusA
SPV-80A_gp25	11801	11986	511	375	348	13121	10854	10075	**11350**	1582.416191	hypothetical protein
SPV-80A_gp26	11987	12346	807	604	509	10706	9033	7614	**9117.666667**	1547.737812	hypothetical protein
SPV-80A_gp27	12347	12595	774	614	501	14846	13276	10835	**12985.66667**	2021.200221	hypothetical protein
SPV-80A_gp28	12610	13011	1061	749	648	12606	10031	8681	**10439.33333**	1994.105898	hypothetical protein
SPV-80A_gp29	13008	13355	880	679	672	12078	10504	10399	**10993.66667**	940.5266255	hypothetical protein
SPV-80A_gp30	13352	13660	388	304	261	5997	5297	4549	**5281**	724.1325845	53 ORF 49-like protei
SPV-80A_gp31	13653	13889	409	285	256	8242	6474	5817	**6844.333333**	1254.199479	hypothetical protein
SPV-80A_gp32	13894	14406	736	630	588	6852	6612	6172	**6545.333333**	344.8671242	dUTPase
SPV-80A_gp33	14443	14649	246	170	161	5676	4421	4188	**4761.666667**	800.3601273	hypothetical protein
SPV-80A_gp34	14646	14849	216	144	148	5057	3800	3907	**4254.666667**	696.8976491	hypothetical protein
SPV-80A_gp35	14842	15078	354	281	258	7134	6383	5862	**6459.666667**	639.4562795	hypothetical protein
SPV-80A_gp36	15071	15457	888	813	645	10959	11310	8975	**10414.66667**	1259.079161	hypothetical protein
SPV-80A_gp37	15454	15627	291	231	187	7988	7147	5788	**6974.333333**	1110.117261	rinB
SPV-80A_gp38	15628	15774	313	243	199	10170	8900	7290	**8786.666667**	1443.341032	rinMiddle
SPV-80A_gp39	15798	16220	1003	841	762	11325	10704	9701	**10576.66667**	819.4536798	rinA
SPV-80A_gp40	16407	16847	640	690	688	6931	8424	8401	**7918.666667**	855.4217284	terminase small subu
SPV-80A_gp41	16768	18111	2279	2895	3002	8099	11597	12028	**10574.66667**	2154.793339	terminase large subu
SPV-80A_gp42	18122	19657	2347	3147	3528	7298	11030	12369	**10232.33333**	2627.920154	portal protein
SPV-80A_gp43	19545	19730	63	66	77	1618	1910	2229	**1919**	305.599411	hypothetical protein
SPV-80A_gp44	19664	20659	1332	1968	2112	6387	10638	11419	**9481.333333**	2708.073916	minor head protein
SPV-80A_gp45	20732	20902	271	345	363	7569	10862	11432	**9954.333333**	2085.326433	hypothetical protein
SPV-80A_gp46	21011	21631	2539	3360	3645	19527	29129	31609	**26755**	6381.268526	scaffold protein

SPV-80A_gp47	21645	22619	3744	4966	5704	18340	27421	31505	**25755.33333**	6738.704648	major head protein
SPV-80A_gp48	22641	22928	568	739	876	9420	13814	16380	**13204.66667**	3519.782001	hypothetical protein
SPV-80A_gp49	22937	23269	796	867	964	11417	14017	15590	**13674.66667**	2107.457315	hypothetical protein
SPV-80A_gp50	23266	23568	1117	1185	1310	17607	21055	23282	**20648**	2859.308133	hypothetical protein
SPV-80A_gp51	23568	23915	1240	1342	1503	17018	20761	23258	**20345.66667**	3140.664951	hypothetical protein
SPV-80A_gp52	23927	24310	1014	1096	1294	12612	15366	18147	**15375**	2767.510976	hypothetical protein
SPV-80A_gp53	24329	24910	1825	1922	2127	14977	17779	19681	**17479**	2366.305982	major tail protein
SPV-80A_gp54	24972	25337	541	639	672	7060	9399	9888	**8782.333333**	1511.490765	hypothetical protein
SPV-80A_gp55	25367	25711	563	627	638	7794	9784	9959	**9179**	1202.632529	hypothetical protein
SPV-80A_gp56	25728	29192	3497	3833	4244	4820	5956	6596	**5790.666667**	899.4694733	tape measure protei
SPV-80A_gp57	27980	28372	188	213	248	2285	2918	3398	**2867**	558.249944	inside tape protein
SPV-80A_gp58	29205	30152	1872	1980	2049	9431	11244	11639	**10771.33333**	1177.444832	hypothetical protein
SPV-80A_gp59	30161	32062	2315	2447	2644	5813	6926	7486	**6741.666667**	851.5963441	hypothetical protein
SPV-80A_gp60	31875	32084	89	78	68	2024	2000	1744	**1922.666667**	155.1945016	hypothetical protein
SPV-80A_gp61	32077	33987	2476	2484	2654	6188	6998	7479	**6888.333333**	652.4494872	minor structure prot
SPV-80A_gp62	33987	35810	1827	1753	1975	4784	5174	5831	**5263**	529.1436478	hypothetical protein
SPV-80A_gp63	35284	35505	48	0	0	1033	0	0	**344.3333333**	596.4028281	hypothetical protein
SPV-80A_gp64	35810	36187	398	299	346	5029	4259	4929	**4739**	418.6884283	hypothetical protein
SPV-80A_gp65	36188	36370	131	109	116	3419	3207	3414	**3346.666667**	120.9807147	hypothetical protein
SPV-80A_gp66	36411	36710	237	241	272	3773	4325	4883	**4327**	555.0027027	hypothetical protein
SPV-80A_gp67	36847	38745	2046	2048	2160	5146	5806	6125	**5692.333333**	499.2998431	lyz
SPV-80A_gp68	38758	39930	1110	1160	1143	4520	5324	5247	**5030.333333**	443.6353608	tail fiber protein
SPV-80A_gp69	39936	40331	599	585	585	7224	7953	7955	**7710.666667**	421.4668828	hypothetical protein
SPV-80A_gp70	40387	40689	216	214	230	3405	3802	4088	**3765**	343	holin
SPV-80A_gp71	40701	42155	1095	1068	1043	3594	3952	3860	**3802**	185.9139586	lyt2
SPV-80A_gp72	42409	42636	3138	5168	5804	65734	122031	137086	**108283.6667**	37610.08982	hypothetical protein
SPV-80A_gp73	42770	43606	13354	12330	10659	76201	79309	68579	**74696.33333**	5520.981917	hypothetical protein
